# Optogenetic restoration of high-sensitivity vision using ChRmine- and ChroME-based channelrhodopsins

**DOI:** 10.1038/s41598-025-04286-9

**Published:** 2025-07-01

**Authors:** Victoria C. Fong, Beatrice M. Le, Antonia Stefanov, Vivian Lee, Seohyun Park, Adithya Sivakumar, Sabrina Spatny, Meike Visel, W. Rowland Taylor, Stephen G. Brohawn, John G. Flannery

**Affiliations:** 1 Herbert Wertheim School of Optometry and Vision Science, The University of California, Berkeley, CA USA; 2 Helen Wills Neuroscience Institute, The University of California, Berkeley, CA USA; 3 Department of Molecular Environmental Biology, The University of California, Berkeley, CA USA; 4 School of Public Health, The University of California, Berkeley, CA USA; 5 Department of Bioengineering, The University of California, Berkeley, CA USA; 6 Department of Molecular and Cell Biology, The University of California, Berkeley, CA USA; 7 Department of Neuroscience, The University of California, Berkeley, CA USA

**Keywords:** Optogenetics, Retinal disease, Vision restoration, Channelrhodopsin, Gene therapy, Mouse behavior, Electrophysiology, Eye diseases, Neuroscience

## Abstract

Optogenetic gene therapy is a promising mutation-independent treatment that aims to restore visual perception in patients blinded by retinal diseases that cause photoreceptor degeneration. Still, low sensitivity or slow kinetics of currently utilized optogenetic proteins limit the efficacy of such approaches. Here, we evaluated the therapeutic potential of three channelrhodopsin variants: ChRmine, from the algae *Rhodomonas lens*, ChRmine-T119A, a faster-closing ChRmine variant, and ChroME2s, a second-generation Chronos-based opsin.We expressed these opsins in retinal ganglion cells of *rd1* mice, a model of severe retinal degeneration. Single cell electrophysiology demonstrates opsin’s large sensitivity to a range of light intensities as well as opsin-expressing retinal ganglion cells generated action potentials in response to light stimulation. Behavioral tests showed ChRmine-T119A’s efficacy at 360 lux compared to unmodified ChRmine and ChroME2s. ChRmine and ChroME2s did restore light perception at higher light intensities. Additionally, our dose–response study with ChRmine-T119A revealed that lower viral titers were more effective at restoring light sensitivity. Our study demonstrates that these ChRmine- and ChroME-based opsins can enhance vision in late-stage blinding diseases.

## Introduction

Degeneration of photoreceptors and the retinal pigment epithelium primarily cause blindness in progressive retinal diseases, including inherited retinal degenerations, age-related macular degenerations and geographic atrophy^1–4^. This degeneration follows a stereotypical progression, with gradual loss of photoreceptors followed by retinal remodeling^4^. However, researchers have observed that inner retinal neurons remain relatively intact in primate and vertebrate models^5,6^, presenting an opportunity for therapeutic intervention.

Despite advances in understanding retinal disease etiology, current treatments are limited for the over 250 disease-causing mutations^1–4^. The sole FDA-approved treatment, Luxturna, for inherited retinal degeneration is applicable only to a small subset of patients with Leber Congenital Amaurosis and is prohibitively expensive in many cases^4^. While more gene therapies are in clinical trials, most target single genes and are only effective in early-stage patients with surviving photoreceptors^1–4^. Alternative approaches, such as stem cell replacements and autosomal dominant genetic editing, face significant challenges and slow progression in clinical trials^2,7^.

Optogenetic gene therapies have emerged as a promising, mutation-agnostic approach to vision restoration. These therapies use microbial light-gated ion channels called channelrhodopsins to impart light sensitivity to surviving retinal neurons^1–3^. However, current synthetic channelrhodopsins in clinical trials, such as ChR2 (NCT02556736), ChrimsonR (NCT03326336), Chronos (NCT04278131), and MCO1 (NCT04919473), have limitations. Their low light sensitivity necessitates the use of light-amplifying goggles to deliver sufficient light intensity for channelrhodopsin activation^8^, subsequently risking phototoxic effects. Conversely, more sensitive variants often have slower kinetics^9,10^, potentially compromising temporal resolution of visual processing.

Recent protein engineering advances have yielded channelrhodopsin variants with improved properties^10–12^. Here, we evaluate the first application of three of the most sensitive and potent channelrhodopsins variants described to date for vision restoration: ChRmine, ChRmine-T119A, and ChroME2s. ChRmine, discovered through structure-based genome mining from *Rhodomonas lens*, displays large photocurrents, broad, red-shifted spectral-sensitivity, and relatively slow kinetics^10,11^. ChRmine-T119A is an engineered variant identified from structure-based design with accelerated closing kinetics (~2-fold in comparison to ChRmine) without reducing photocurrent magnitudes or sensitivity^12^. ChroME2s is a second-generation variant of the parental Chronos opsin from the algae *S. helveticum* that shows substantially increased photocurrents with only modestly slower kinetics^10^. These opsins’ enhanced performance *in vitro, in vivo,* and *in situ* suggest potential for vision restoration under standard indoor lighting conditions^10–12^.

In this study, we leveraged the advantageous characteristics of these opsins to develop highly sensitive optogenetic gene therapies capable of restoring visual function in a mouse model of retinal degeneration. Additionally, this study explores the importance of viral titer in influencing the efficacy of the optogenetic treatment and light-sensitivity outcomes.

## Results

### Opsin expression and localization in retinal ganglion cells

**Fig. 1:**
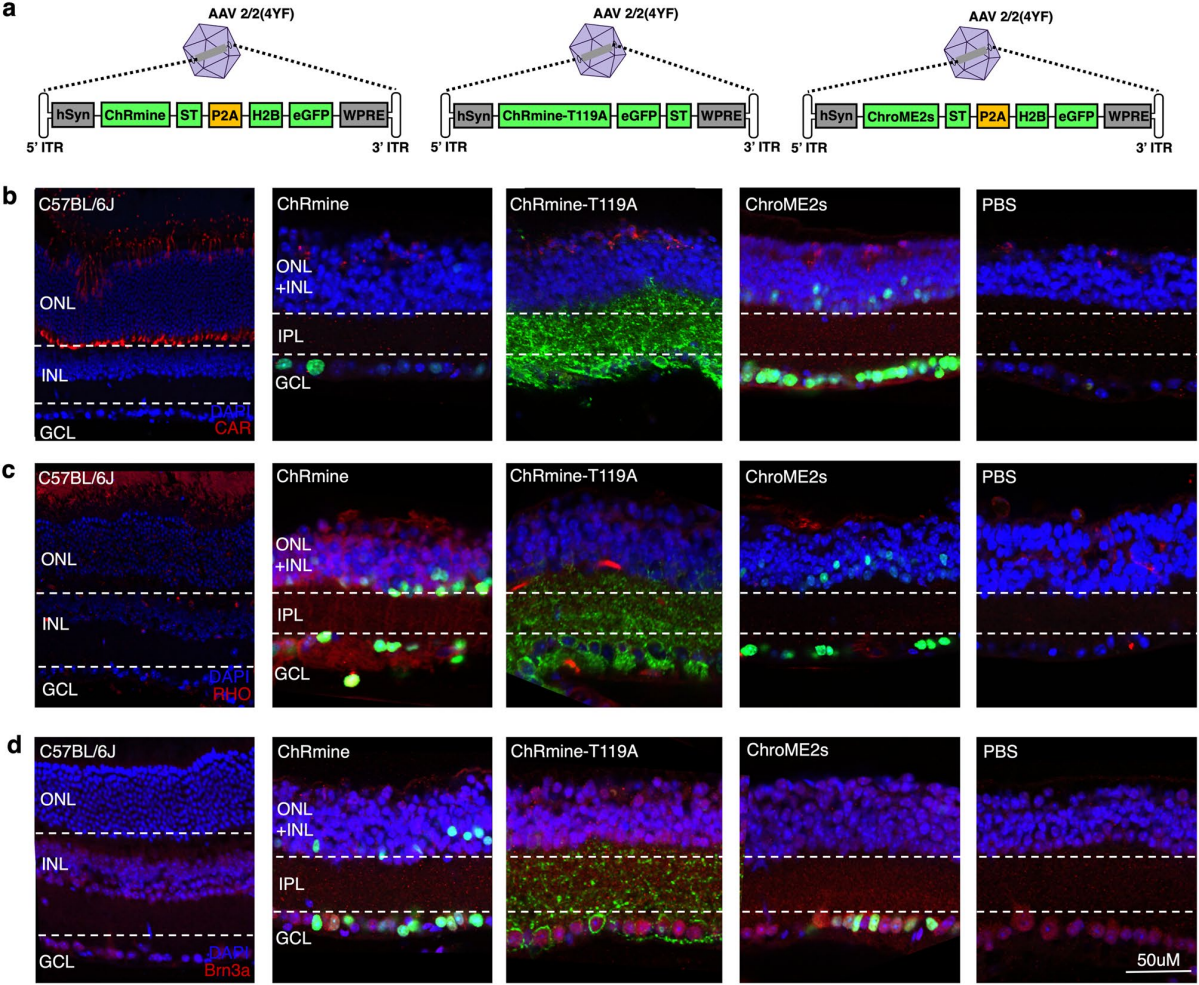
Immunohistochemistry of retinas treated with ChRmine, ChRmine-T119A, and ChroME2s AAVs. (**a**) Schematic of the AAV transgene cassettes. (**b-d**) Immunofluorescence stained-images of retinal sections. eGFP represents opsin expression in treated *rd1* mice. Cone photoreceptors are labeled using CAR (**b, red**) and rod photoreceptors with RHO (**c, red**). (**d**) Co-localization of the opsin (eGFP) and Brn3a (red) confirm expression in ganglion cells. CAR = cone arrestin, RHO = rhodopsin, eGFP = enhanced green fluorescent protein.

We evaluated the potential efficacy of these opsins by expressing them in the retinas of *rd1* mice, a retinal degeneration model with significant loss of photoreceptors^13,14^. We assessed retinal transduction at least 8 weeks post-intravitreal injection of a recombinant AAV2/2(4YF) viral vector. All AAV constructs contained a soma targeting (ST) motif to enhance soma localization and clustering of the opsins to the cell somas^15^. Opsin expression was driven by the human synapsin promoter (*hSyn*), a commonly used neuron-specific promoter with preferential expression in retinal ganglion cells (RGCs)^16^. To visualize transduced cells, we included enhanced green fluorescent protein (eGFP) in our vectors, either directly fused to the channelrhodopsin or as a separate histone 2B (H2B) fusion protein following a P2A ribosomal skipping sequence^17,18^ (Fig. 1a).

Immunohistochemistry performed on *C57Bl/6*J and *rd1* retinas confirmed successful transgene expression, in both RGCs and interneurons (Figs. 1b-d). In *C57Bl/6J* retinas, we confirmed that 55.2 ± 10% of eGFP+ cells were RGCs in ChRmine-treated mice, 58.2 ± 6 % in ChRmine-T119A-treated mice, and 56.2± 6% in ChroME2s-treated mice (Supplemental Fig. S1) The localization of eGFP varied between constructs due to the differences in fusion strategy. In ChRmine-T119A-treated retinas, eGFP localizes to the cell membrane and dendrites of neurons. In contrast, ChRmine and ChroME2s-transfected cells showed soma-localization of eGFP, reflecting the separate H2B-eGFP expression in these constructs (Supplementary Fig. S2)

### Electrophysiological characterization of opsin-expressing retinal ganglion cells

Recent studies have shown that ChRmine, ChRmine-T119A, and ChroME2s exhibit larger photocurrents compared to many opsins currently in use^10–12^. However, channel properties have only been directly characterized in cultured cells *in vitro*^10–12^ and brain neurons^10,19^. To address this gap, we performed *ex vivo* single-cell electrophysiology on transduced RGCs.

**Fig. 2:**
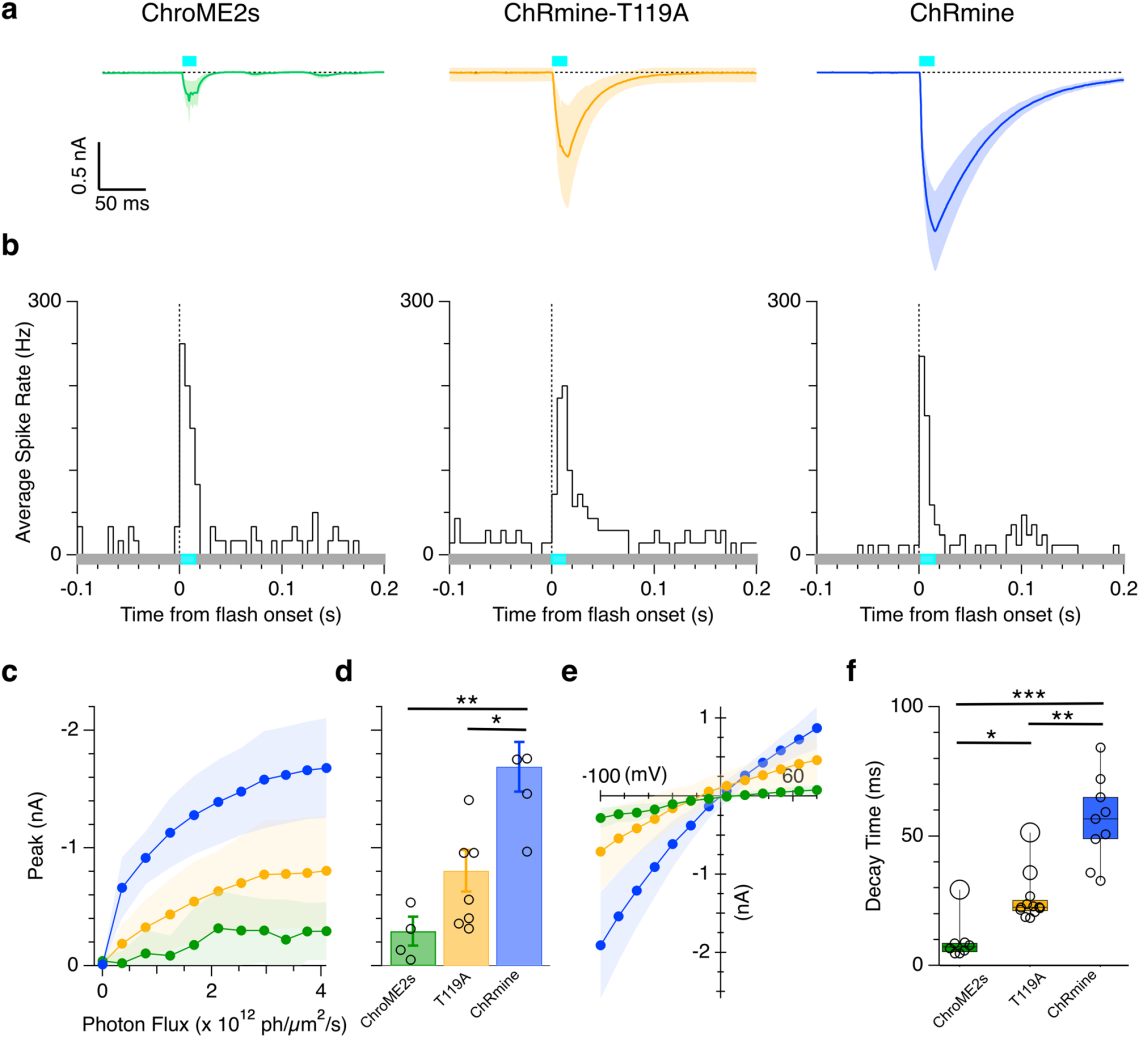
Characterization of RGCs expressing ChroME2s, ChRmine-T119A, and ChRmine opsins in response to blue LED light flashes. (**A**) Average ChroME2s (n = 3), ChRmine (n = 4), and ChRmine-T119A (n = 4 cells) mediated photocurrents in RGCs in response to diffuse 485 nm light flash of 167 mW/cm2 (timing shown by the cyan bar). (**B**) Average peristimulus spike-time histograms (PSTH) generated from ChroME2s- (200.0 ± 102.5 Hz, n = 12 cells), ChRmine-T119A- (152.4 ± 144.2 Hz, *n = 14 cells*), and ChRmine- (152.9 ± 84.2 Hz during stimulus, *n = 17 cells*) expressing RGCs. Light flash was 32.2 mW/cm^2^ for 15 ms. (**C**) Average peak photocurrent as a function of flash intensity. (**D**) Max peak photocurrent at the highest light intensity for ChRmine- (1.69 ± 0.42 nA, *n = 4 cells*), ChRmine-T119A- (0.81 ± 0.47 nA, *n = 7 cells*), and ChroME2s-expressing RGCs (0.30 ± 0.24 nA, *n = 4 cells*). Error bars in bar graph are represented as SEM (**E**) Current–voltage relations for the peak photocurrents of ChroME2s- (*n = 36 cells*), ChRmine-T119A- (*n = 7 cell*s), and ChRmine-expressing RGCs (*n = 6 cells*). (**E**) Exponential decay time constants measured from the photocurrents in ChroME2s- (9.5 ± 8.2, *n = 8 cells*), ChRmine-T119A- (25.6 ± 9.3 ms, *n = 12 cells*), and ChRmine-expressing RGCs (56.2 ± 16.5 ms, *n = 9 cells*). *p < 0.05, **p < 0.01, and ***p < 0.001. Shading shows the standard deviation in all panels.

To assess the functional expression of these opsins in RGCs, we measured membrane currents, spiking responses, opsin decay kinetics, and current-voltage relations. We voltage-clamped RGCs displaying eGFP fluorescence and measured photocurrents in response to 15 ms flashes of diffuse 485 nm light (Fig. 2a, Supplemental Fig. S3). Onset latencies were around 1.6 to 2 ms (Supplemental Fig. S6) and the decay time-constant (Fig. 2f) aligned well with previously reports^10–12^. We measured current-voltage relations for currents elicited by a fixed light intensity at holding potentials from −90 to +60 mV. Current-voltage relations reversed close to zero millivolts and displayed mild inward rectification, similar to the previous reports (Fig. 2e).

We measured photocurrents at a range of light intensities from 0.13 to 167.1 mW/cm^2, to estimate the maximum amplitude of the opsin-mediated currents. Peak photocurrents ranged from several hundred picoamperes to about 2 nA (Fig. 2c-d). These differences likely result from variation in the single-channel currents, and potentially systematic differences in the expression levels among the different variants. To isolate ChR contributions to RGC activity, we pharmacologically blocked postsynaptic ionotropic glutamate receptors using D-AP5 and NBQX (Supplemental Fig. S4). Light-evoked photocurrent persists under a combined D-AP5 and NBQX application, with waveform characteristics remaining consistent with controlled conditions.

Given that RGCs typically have input resistances of 100 to several hundred megaohms^20^, even the smaller ChroME2s photocurrents of a few hundred picoamperes should sufficiently depolarize a RGC to reach its spike threshold. To confirm this functional efficacy, we recorded extracellular spiking responses. Spike-time histograms generated from ChroME2s, ChRmine-T119A, and ChRmine (Fig. 2b, Supplemental Fig. S5) displayed the short latencies (~1.6 ms for ChroME2s and 2 ms for ChRmine; Supplementary Fig. S6) and transient responses expected from the temporal properties of the opsin-mediated photocurrents.

### ChRmine and ChRmine-T119A restore innate aversion to light

Sighted mice are biologically programmed to avoid brightly illuminated spaces, preferring dimmer areas^21^. This innate behavior is lost in *rd1* mice as a result of retinal degeneration. To assess if our candidate opsins could restore this behavior, we employed a passive light avoidance test using a shuttle box with separate dark and illuminated chambers connected by a door. We first conducted a 15-minute habituation period in complete darkness to account for potential biases in compartment preference. These biases can arise from various factors including associating one side with shelter, exhibiting freezing or fleeing behavior due to fear in a novel environment, avoiding a compartment due to unfamiliarity with the experimenter, or general stress^22,23^.

**Fig. 3:**
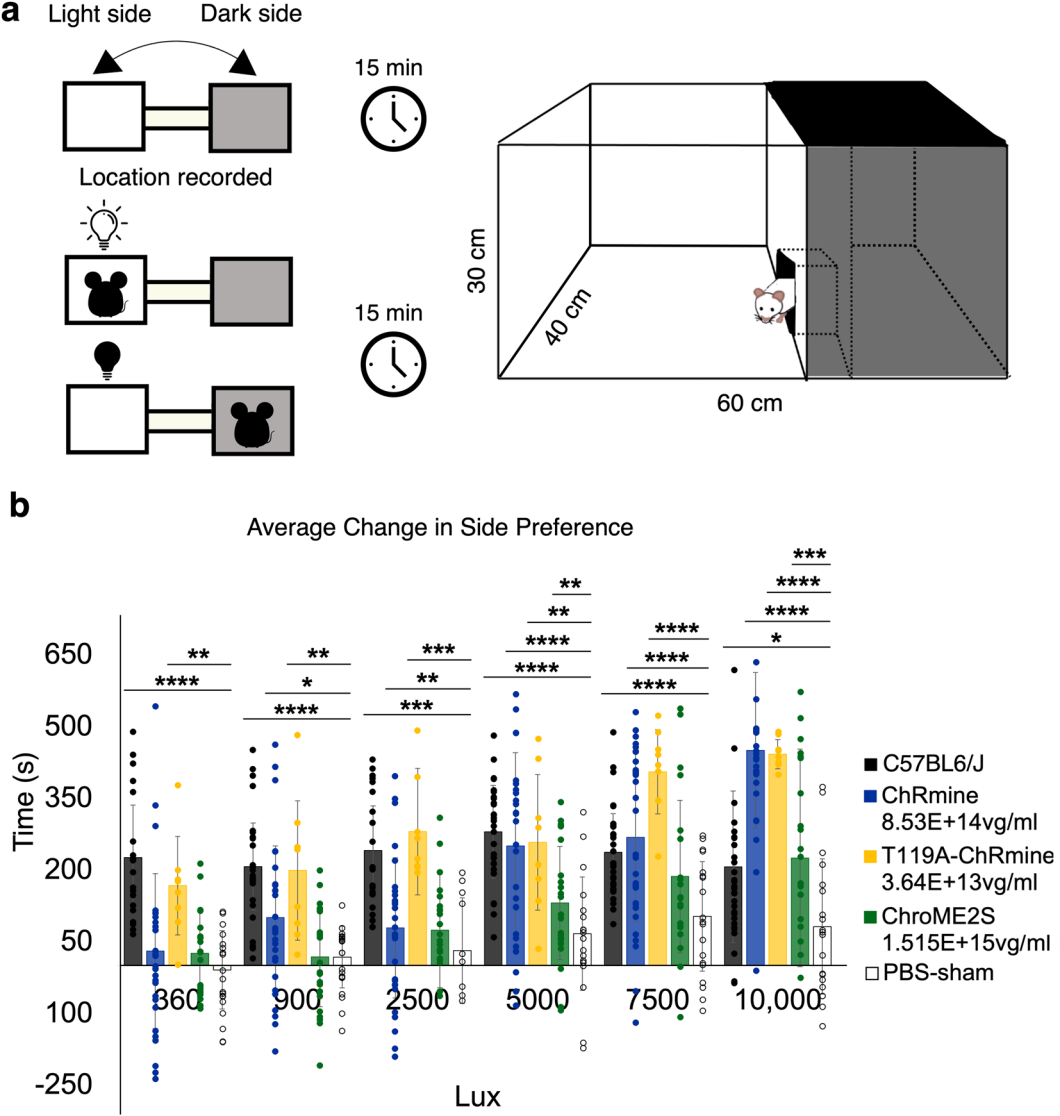
Light avoidance experiments in *rd1* mice expressing ChRmine, ChRmine-T119A, or ChroME2s. (**a**) Schematic of light/dark box for light avoidance test based on Riebe and Wotjak, 2012^24^. **(b)** Average change in side preference using white light (irradiance of 100 μW cm^−2^) in *C57BL6/J* (*n = 26*), ChRmine- (*n = 29*), ChRmine-T119A- (*n = 8*), ChroME2s- (*n = 21*), and PBS- (n = 19) injected mice. Statistical significance assessed with Welch’s ANOVA test with post-hoc Games-Howell HSD threshold matrix, *p < 0.05, **p < 0.01, ***p < 0.0001.

Following habituation, we activated an LED-floodlight that illuminated the mouse’s preferred compartment when movement was detected and then turned off when the mouse moved to the adjoining, unpreferred compartment. This setup trained mice to avoid their biased locations if they could perceive light during a 15-minute experimental trial (Fig. 3a).

We measured change in side preference, which is defined as the change in time an animal spends on its initially preferred side (determined during the habituation trial) between habituation and the experimental trial. A positive value indicates that the animal spent less time on its previously preferred side during the experimental trial, suggesting light avoidance. A negative value indicates that the animal spent more time on the initially preferred side during the experimental trial, implying that light had no effect on avoidance behavior. Light thresholds were further measured using lux, a unit of illuminance. The lowest activation thresholds were determined by comparing behavior with PBS-injected controls.

Treated mice show significant rescue of light avoidant behavior. The light threshold of ChRmine-treated mice was 900 lux (*p*=0.012, 100.63 ± 149.76 s), ChRmine-T119A-treated mice was 360 lux (*p*=*0.002*, 167.52 ± 102.985 s), and ChroME2s-treated mice was 5000 lux (*p=0.009*, 131.35 ± 118.33 s) (Fig. 3b). These results align with our previous findings in RGCs (Fig. 2) and reflect earlier research^10–12^.

To assess spectral sensitivity, we tested ChRmine (peak excitation: ~550 nm) and ChroME2s (peak excitation: ~500 nm) expressing *rd1* mice with green (535 nm) and red (610 nm) light. Contrary to expectations based on CHO cell studies^10–12^, our behavioral tests showed poor responses at the tested intensities (Supplemental Fig. S7).

### Lower titers of ChRmine-T119A are more effective in restoring light sensitivity

**Fig. 4:**
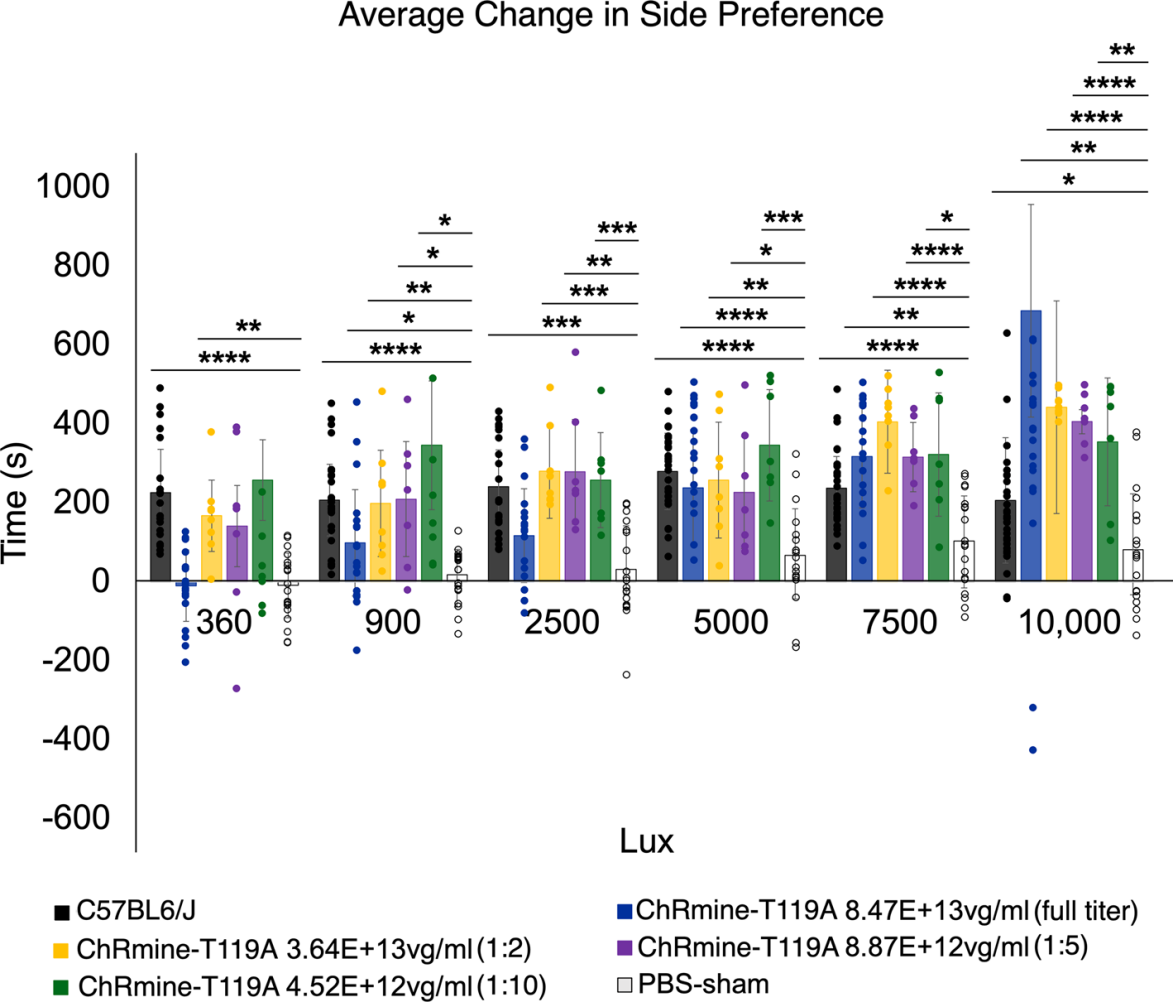
Light aversion experiments in *rd1* mice treated with different titers of ChRmine-T119A. Mice treated with lower titers are more light-sensitive than mice treated with the highest titer. Average change in side preference using white light (irradiance of 100 μW cm^−2^). *C57BL/6J* (n*=26*), ChRmine-T119A undiluted (*n = 18*), ChRmine-T119A 1:2 (*n = 8*), ChRmine-T119A 1:5 (*n = 7*), ChRmine-T119A 1:10 (*n = 7*), and PBS (*n = 19*). Data represents the mean ± SD. Statistical significance assessed with Welch’s ANOVA test with post-hoc Games-Howell HSD threshold matrix, *p < 0.05, **p < 0.01, ***p < 0.0001.

To investigate potential excitotoxicity and optimize treatment efficacy, we conducted a dose-response study using four different titers of ChRmine-T119A (Methods, Table 1). In the light avoidance test, mice treated with a lower titer (1:2; 3.64E+13 vg/mL) showed greater light sensitivity at 360 lux than those treated with the highest titer (undiluted; 8.47E+14 vg/mL) (*p*=0.974, −9.68 ± 91.59 s (SD)). These results are plotted in Figure 4. While the highest titer showed significant light avoidance compared to PBS-treated controls at 10,000 lux (*p*=0.005, 301.82 ± 269.51 s), 7,500 lux (*p*=1.315E-6, 317.79 ± 130.52 s), 5,000 lux (*p*=5.731E-5, 238.32 ± 152.96 s), and 900 lux (*p*=0.047, 98.72 ± 149.93 s), the lower titer groups maintained a more significant light avoidance at 10,000 lux (1:2 *p*=7.638E-6, 442.57 ± 122.68 s ; 1:5 *p*=4.272E-9, 406.08 ± 33.07 s ; 1:10 *p*=0.003, 354.71 ± 162.07 s), 2,500 lux (1:2 *p*=0.0001, 303.14 ± 120.34 s ; 1:5 *p*=0.004, 278.97 ± 153.25 s ; 1:10 *p*=0.0001, 238.83 ± 85.87 s), 900 lux (1:2 *p*=0.01, 199.09 ± 145.67 s ; 1:5 *p*=0.03, 205.41 ± 170.9 s ; 1:10 *p*=0.029, 199.47 ± 162.75 s), and 360 lux (1:2 *p*=0.002, 167.52 ± 102.98 s). These findings suggest that lower titers of ChRmine-T119A may be more effective in restoring light sensitivity, potentially due to reduced excitotoxicity. Further research is needed to fully understand the relationship between titer, efficacy, and potential side effects.

**Table 1:** A list of AAVs produced and their titers. Vg = viral genomes.

AAV	Titer (vg/mL)
2-4YF hSyn ChRmine ST P2A H2B eGFP WPRE	8.53E + 14
2-4YF hSyn ChRmine-T119A eGFP ST WPRE	8.47E + 13
2-4YF hSyn ChRmine-T119A eGFP ST WPRE (1:2)	3.64E + 13
2-4YF hSyn ChRmine-T119A eGFP ST WPRE (1:5)	8.87E + 12
2-4YF hSyn ChRmine-T119A eGFP ST WPRE (1:10)	4.52E + 12
2-4YF hSyn ChroME2s ST P2 A H2B eGFP WPRE	1.52E + 15

## Discussion

Clinical trials using optogenetic gene therapy have demonstrated recovery of light perception, but there remains space for substantial improvement. While results are extremely promising, we investigated whether more recently characterized channelrhodopsins with improved functional properties could offer benefits. Our study demonstrates the potential of three novel channelrhodopsin variants, ChRmine, ChRmine-T119A, and ChroME2s for AAV-based optogenetic vision restoration in a mouse model of retinal degeneration. Our findings revealed that these opsins can restore visual function to varying degrees, with ChRmine-T119A showing particular promise for its efficacy at indoor light levels.

Electrophysiological characterization of opsin-expressing RGCs confirmed the high light sensitivity of these opsins in the retina, with properties in concordance with previous reports in CHO cells^10–12^ and mouse brain slices^10^. The light avoidance behavioral test revealed that ChRmine-, ChRmine-T119A-, and ChroME2s-treated mice were sensitive to a range of light intensities. Notability, ChRmine-T119A demonstrated light sensitivity at room light levels (360 lux), a significant improvement over current optogenetic approaches in clinical trials. To contextualize these light activation thresholds with natural lighting conditions, the intensity threshold of ChRmine-treated *rd1* mice is just below an overcast day, ChRmine-T119A mice is at around office lighting, and ChroME2s-ST mice is at full daylight^25^ (Supplementary Fig. S8).

A notable discrepancy exists between out *ex vivo* electrophysiological recordings and *in vivo* behavior results: ChRmine-expressing mice exhibited lower light activation thresholds *in vivo* compared to ChRmine-T119A counterparts, despite generating larger photocurrents *ex vivo*. We selectively targeted GFP+ cells, therefore the *ex vivo* magnitude does not fully predict *in vivo* efficacy. It is possible that the variable expression of hSyn promoter-driven vectors in RGCs could be attributed to the functional and molecular heterogeneity of RGCs, or transduction efficiency of the different vector elements.

Unexpectedly, we observed poor responses to green (535 nm) and red (610 nm) light in ChRmine- and ChroME2s-expressing *rd1* mice, despite previous reports^10^ of sensitivity to these wavelengths in other systems. This discrepancy may be due to factors such as light absorption, scatter, and chromatic aberration in the eye^26^, highlighting the importance of *in vivo* behavioral testing for optogenetic therapies.

Our dose-response study with ChRmine-T119A revealed that lower viral titers were more effective at restoring light sensitivity. We did not conduct dose-response experiments for ChRmine or ChroME2s opsins, but it is possible that lower doses of ChRmine and ChroME2s may be more effective than the doses tested in this study. This finding raises important questions about potential excitotoxicity at higher expression levels and emphasizes the need for careful dose optimization in future clinical applications.

A limitation of our study was the pan-neuronal expression of our opsins^16,27,28^. When we transfected our AAV constructs in *C57Bl6/J* mice, we found widespread eGFP expression in RGCs and likely displaced amacrine cells (Supplementary Fig. S1). Future treatments should consider using a more cell-type specific promoter than *hSyn.* To understand the effect of opsins without the interference of other neuronal signaling in the retina, expression must be cell-type specific with minimal off-target transduction.

In conclusion, our results demonstrate that advancements in opsin engineering directly translate to improvements in optogenetic vision restoration. The enhanced light sensitivity of these new opsins, particularly ChRmine-T119A, offer promising avenues for developing more effective treatments for late-stage retinal degeneration. Future work should focus on optimizing delivery methods, refining retinal cell-type targeting, and further exploring the relationship between opsin properties and functional visual outcomes.

## Methods

### rAAV production

Transgene plasmids containing the human synapsin (*hSyn-1*) promoter upstream of the opsin of interest, flanked by ITR sites, were engineered. These plasmids were co-transfected with plasmids containing the *rep* and *cap* genes for the AAV2/2(4YF) capsid and plasmids containing necessary adenovirus genes for AAV replication (E4, E2a, VA) into the HEK 293T mammalian packaging cell line. After 2–3 days of incubation to allow for the production of virus particles, the cells were harvested and lysed. AAVs were purified using iodixanol gradient centrifugation. Viral titers were quantified via qPCR with primers for ITR sites. The vectors were then aliquoted and masked externally.

### Animals and intravitreal injection

All animal procedures were approved by the Institutional Animal Care and Use Committee of the University of California, Berkeley. Mice experiments were conducted in accordance with the ARVO Statement for the Use of Animals in Ophthalmic and Vision Research. All animal procedures also abide by all Arrive guidelines as started: https://arriveguidelines.org.

*Rd1* mice (*C3H/HeJ*) and *C57BL/6J* mice were purchased from the Jackson Laboratory and housed on a 12-h light/dark cycle. Mice were anesthetized with intraperitoneal ketamine (100 mg/kg) and xylazine (10 mg/kg). Eyes were anesthetized with proparacaine (0.5%), and pupils were dilated with phenylephrine (2.5%) and tropicamide (1%). At postnatal day 90, the vectors were intravitreally injected at the ora serrata using a 30-gauge needle and 2 μL of rAAV was deposited in the vitreous of the mouse eye with a Hamilton 80000 1701N microliter syringe.

### Tissue preparation for immunohistochemistry

Mice >4 weeks post-treatment were sacrificed, eyes were fixed overnight in 4% paraformaldehyde (Electron Microscopy Sciences, PA, USA). Retinas were dissected from sclera and anterior anatomical parts and thoroughly washed with phosphate buffer saline (PBS; Thermo Fisher Scientific, MA, USA). Whole mounts were embedded in 5% low-melting point agarose (Fisher Scientific, Hampton, NH) and sectioned transverse at a 100 μm thickness using a vibratome (VT1000S, Leica Microsystems, CA, USA).

### Immunohistochemistry and imaging

Whole mounted retinas were incubated in a blocking buffer (0.025% NaN_3_, 1% Triton-X, 10% Normal Donkey serum in PBS) for an hour. Sample was then incubated in Primary antibody solution (0.025% NaN3, 1% Triton-X, 3% Normal Donkey serum, PBS, 1:200 rabbit anti-RBPMS (Thermofisher, PA5-31231) in PBS) overnight. After PBS washes, sections were incubated with Alexa Fluor 647 donkey anti-rabbit (Thermofisher, A31573, 1:800) for 2 hours. Samples were washed and mounted on slides using Vectashield Antifade Mounting Medium with DAPI (Vector Laboratories).

Agarose-embedded slices were incubated in a blocking buffer (0.1% NaN_3_, 10% Triton-X, 5% BSA, 5% normal goat serum in PBS) overnight. Primary antibodies were applied overnight with the following dilutions: Rabbit anti-cone arrestin (EMD Millipore, AB15282, 1:1,000), mouse anti-rhodopsin (EMD Millipore, MABN15, 1:750), or rabbit anti-Brn3a (EMD Millipore, AB5945, 1:400 or Millipore Sigma, MAB1585, 1:500). After a PBS wash, sections were incubated with Alexa Fluor 594 goat anti-rabbit (Life Technologies, A-1102, 1:1,000), Alexa Fluor 594 goat anti-mouse (Life Technologies, A-11032, 1:1,000), or Alexa Fluor 488 goat anti-mouse (Invitrogen, A-11001, 1:1000) overnight. Sections were mounted on slides using Vectashield Antifade Mounting Medium with DAPI (Vector Laboratories). All incubation times and washes were at 4 °C. Samples were processed and examined by confocal laser scanning microscopy (LSM 880, Carl Zeiss, Oberkochen, Germany).

### Passive light avoidance task

A two-compartment shuttle box (Colbourn Instruments, USA) was used. Mice habituated for 15 minutes in the dark, and a floodlight was attached to their preferred chamber. A 15-minute trial began when mice crossed into their preferred chamber. Light intensities of 10000, 7500, 5000, 2500, 900, or 360 lux intensity were tested. Animals were tracked using sensors at the base of the shuttle box. Time spent in the dark and change in side preference was recorded and analyzed using the Graphic State RT programs from Colbourn Instruments.

### Behavioral analysis

Behavioral tests were performed while the experimenter was masked to all cohorts except for *C57BL/6J* positive control mice. Treated and PBS-sham injected mice, however, remained blind to the experimenter. We show change in side preference, which is defined as the change in time an animal spends on its initially preferred side (determined during the habituation trial) between habituation and the experimental trial. A positive value indicates that the animal spent less time on its previously preferred side during the experimental trial, suggesting light avoidance. A negative value indicates that the animal spent more time on the initially preferred side during the experimental trial, implying that light had no effect on avoidance behavior. In Supplementary Figure S7, we also show the amount of time spent in the dark during the experimental trial.

### Tissue preparation for electrophysiology

Experimental mice were dark-adapted for at least 1 hour prior to euthanasia. Subsequent animal handling after dark-adaptation was performed in dim red light. Mice was euthanized in an isoflurane drop jar followed by cervical dislocation. Bicarbonate-buffered AMES media was prepared by combining AMES with L-Glutamine (US Biological Life Sciences, #A1372-25) with Sodium bicarbonate (Fisher Bioreagents, #BP328).

Eyes were enucleated and submerged in bicarbonate-buffered AMES media with L-Glutamine which was oxygenated with 95% O2 and 5% CO2 gas formulation. The anterior portions of the eye, vitreous humor, and retinal pigment epithelial layer were dissected from the retina under infrared illuminated microscope ocular lens (~900 nm). The retina was prepared with four relief radial cuts and mounted onto an anodisc (Whatman, #6809-7023). The mounted retina was placed into a recording chamber, ganglion cell side up, which was perfused with oxygenated bicarbonate-buffered AMES media.

### Electrophysiological recording

Micropipettes were fabricated from Borosilicate glass filaments on a vertical puller (Sutter Instrument, BF150-86-7.5). Extracellular solution contained bicarbonate-buffered AMES media with L-Glutamine filtered with a 0.2 μm filter (Nalgene, 171-0020). Micropipettes for extracellular recordings have a resistance around (4–8 Ohms) and are filled with filtered AMES solution. Micropipettes for intracellular recordings have resistance around (4–7 Ohms). These micropipettes were filled with a solution containing 6 mM CsMeSO4, 110 mM CsCl, 10 mM NaCl, 525 μM Na-HEPES, 1 mM Cs-EGTA, 1 mM Na-ATP, Alexa 594 hydrazide (50–100 μM), 0.1 mM Na-GTP, and 10 mM QX-314. The QX-314 and Cs^+^ -gluconate was used to block voltage-dependent sodium channels and block outward rectifier potassium channels.

Dye was added to the intracellular solutions for morphological identification of recorded cells. The liquid junction potential was accounted for by adjusting the membrane potential by −10 mV. Cell somas were visualized under infrared illumination (940 nm) through a KT&C CCD Camera Hi-Res EXvision camera and LUMPlanFl 40x objective. Somas were loose-cell patched and flashed with 15 ms of a diffused 485 nm light.

The initial baseline current has been subtracted from each trace to compare the amplitudes. Cells were voltage-clamped at resting potential (−60 mV) and flashed with 15 ms pulses of blue LED light with different intensities. Light intensity was controlled by modifying the voltage of the light source and calculated irradiance based on power measurements from a ThorLabs photosensor device. Voltage-clamp measurements for current-voltage relations were performed by applying voltages ranging from −100 to +80 mV with a step size of 15 mV and a holding time at each potential of 1.0 sec. The holding potential between each voltage step is −60 mV. Then, the retina was pulsed with a 15 ms blue LED light flash with an irradiance of 68.5 mW/cm^2. All kinetic analyses were performed with custom Igor 9.0 routines. Peak responses, decay components, and intensity-response values were averaged individually for each RGCs. Decay rates were fitted with a single exponential function and values were plotted using Tukey outlier and quartile method; Larger dot size corresponds to a farther outlier. Supplemental Figure S4 contains traces before and after application of D-AP5 (50 μM) and NBQX (25 μM).

### Statistical analysis

Figure 2c and Supplemental Figure S7 were analyzed using ANOVA and Tukey. Figure 2e data was analyzed using Kruskal-Wallis and Mann-Whitney U-Test for pairwise comparisons. Significance levels were adjusted by applying Bonferroni corrections.

To evaluate the effects of treatment across different illuminance levels in our behavioral experiments, we conducted Welch’s ANOVA test with post-hoc Games-Howell HSD threshold matrix, comparing treatment and control groups at each illuminance level independently. This method was selected for its robustness to violations of normality and unequal sample sizes.

Data is presented as mean ± SD unless otherwise stated. Differences were considered statistically significant if the p-value was less than 0.05. In the figures, p-values < 0.05 are indicated with a single asterisk (*), p-values < 0.01 are indicated with a double asterisk (**), p-values < 0.001 are indicated with a triple asterisk (***), and p-values < 0.0001 are indicated with a quadruple asterisk (****).

## Supplementary Information

Supplementary Information 1.

Supplementary Information 2.

Supplementary Information 3.

Supplementary Information 4.

Supplementary Information 5.

Supplementary Information 6.

Supplementary Information 7.

Supplementary Information 8.

## Data Availability

All data generated or analyzed during this study will be made available by the authors under reasonable request.

## References

[1] Ku, C. A. & Pennesi, M. E. The new landscape of retinal gene therapy. *Am. J. Med. Genet. C Semin. Med. Genet.* **184**, 846–859 (2020). 32888388 10.1002/ajmg.c.31842

[2] Botto, C. et al. Early and late stage gene therapy interventions for inherited retinal degenerations. *Prog. Retin. Eye Res.* **86**, 100975 (2022). 34058340 10.1016/j.preteyeres.2021.100975

[3] Stefanov, A. & Flannery, J. G. A systematic review of optogenetic vision restoration: History, challenges, and new inventions from bench to bedside. *Cold Spring. Harb. Perspect. Med.* **13**, a041154 (2023). 10.1101/cshperspect.a041304 PMC10234433 36376079

[4] Pfeiffer, R. L. & Jones, B. W. Current perspective on retinal remodeling: Implications for therapeutics. *Front. Neuroanat.* **16**, 1099348 (2022). 36620193 10.3389/fnana.2022.1099348 PMC9813390

[5] Puthussery, T. & Taylor, W. R. Functional changes in inner retinal neurons in animal models of photoreceptor degeneration. *Adv. Exp. Med. Biol.* **664**, 525–532 (2010). 20238055 10.1007/978-1-4419-1399-9_60

[6] Mazzoni, F., Novelli, E. & Strettoi, E. Retinal ganglion cells survive and maintain normal dendritic morphology in a mouse model of inherited photoreceptor degeneration. *J. Neurosci.* **28**, 14282–14292 (2008). 19109509 10.1523/JNEUROSCI.4968-08.2008 PMC2633359

[7] Barker, R. A. et al. The challenges of first-in-human stem cell clinical trials: What does this mean for ethics and institutional review boards?. *Stem. Cell Rep.* **10**, 1429–1431 (2018). 10.1016/j.stemcr.2018.04.010 PMC5995446 29742388

[8] Sahel, J. A. et al. Partial recovery of visual function in a blind patient after optogenetic therapy. *Nat. Med.* **27**, 1223–1229 (2021). 34031601 10.1038/s41591-021-01351-4

[9] Pan, Z. H., Ganjawala, T. H., Lu, Q., Ivanova, E. & Zhang, Z. ChR2 mutants at L132 and T159 with improved operational light sensitivity for vision restoration. *PLoS ONE* **9**, e98924 (2014). 24901492 10.1371/journal.pone.0098924 PMC4047080

[10] Sridharan, S. et al. High-performance microbial opsins for spatially and temporally precise perturbations of large neuronal networks. *Neuron* **110**, 1139-1155.e6 (2022). 35120626 10.1016/j.neuron.2022.01.008 PMC8989680

[11] Bansal, H., Pyari, G. & Roy, S. Theoretical prediction of broadband ambient light optogenetic vision restoration with ChRmine and its mutants. *Sci. Rep.* **14**, 11642 (2024). 38773346 10.1038/s41598-024-62558-2 PMC11109128

[12] Tucker, K., Sridharan, S., Adesnik, H. & Brohawn, S. G. Cryo-EM structures of the channelrhodopsin ChRmine in lipid nanodiscs. *Nat. Commun.* **13**, 4842 (2022). 35977941 10.1038/s41467-022-32441-7 PMC9385719

[13] LaVail, M. M. & Sidman, R. L. C57BL/6J Mice with inherited retinal degeneration. *Arch. Ophthalmol.* **91**, 394–400 (1974). 4595403 10.1001/archopht.1974.03900060406015

[14] Zhang, L. et al. The temporal progression of retinal degeneration and early-stage idebenone treatment in the Pde6b rd1/rd1 mouse model of retinal dystrophy. *Sci. Rep.* **14**, 2019 (2024). 38263197 10.1038/s41598-024-52391-y PMC10805728

[15] Lim, S. T., Antonucci, D. E., Scannevin, R. H. & Trimmer, J. S. A. Novel targeting signal for proximal clustering of the Kv2.1 K+ channel in hippocampal neurons. *Neuron* **25**(385), 397 (2000). 10.1016/s0896-6273(00)80902-2 10719893

[16] Nieuwenhuis, B. et al. Improving adeno-associated viral (AAV) vector-mediated transgene expression in retinal ganglion cells: Comparison of five promoters. *Gene. Ther.* **30**, 1–17 (2023). 36635457 10.1038/s41434-022-00380-z PMC10284706

[17] Kim, J. H. et al. High cleavage efficiency of a 2A peptide derived from porcine teschovirus-1 in human cell lines, zebrafish mice. *PLoS ONE* **6**, e18556 (2011). 21602908 10.1371/journal.pone.0018556 PMC3084703

[18] Luo, A. et al. H2B ubiquitination recruits FACT to maintain a stable altered nucleosome state for transcriptional activation. *Nat. Commun.* **14**, 741 (2023). 36765085 10.1038/s41467-023-36467-3 PMC9918737

[19] Kishi, K. E. et al. Structural basis for channel conduction in the pump-like channelrhodopsin ChRmine. *Cell* **185**, 672–689 (2022). 35114111 10.1016/j.cell.2022.01.007 PMC7612760

[20] Wang, G. et al. The sensitivity of light-evoked responses of retinal ganglion cells is decreased in nitric oxide synthase gene knockout mice. *J. Vis.* **7**, 7 (2007). 10.1167/7.14.7 18217802

[21] Bourin, M. & Hascoët, M. The mouse light/dark box test. *Eur. J. Pharmacol.* **463**, 55–65 (2003). 12600702 10.1016/s0014-2999(03)01274-3

[22] Segelcke, D. et al. Experimenter familiarization is a crucial prerequisite for assessing behavioral outcomes and reducing stress in mice not only under chronic pain conditions. *Sci. Rep.* **13**, 2289 (2023). 36759654 10.1038/s41598-023-29052-7 PMC9911644

[23] Gouveia, K. & Hurst, J. L. Improving the practicality of using non-aversive handling methods to reduce background stress and anxiety in laboratory mice. *Sci. Rep.* **9**, 20305 (2019). 31889107 10.1038/s41598-019-56860-7 PMC6937263

[24] Riebe, C. J. & Wotjak, C. T. A Practical Guide to Evaluating Anxiety-Related Behavior in Rodents. In *TRP Channels in Drug Discovery: Volume II* (eds. Szallasi, A. & Bíró, T.) 167–185 (Humana Press, Totowa, NJ, 2012).

[25] Mohamed, S. B. et al. An intelligent lighting control system (ILCS) using LabVIEW. *J. Fundam. Appl. Sci.* **9**, 602–615 (2017).

[26] Ruddock, K. H. Light Transmission through the Ocular Media and Macular Pigment and its Significance for Psychophysical Investigation. In *Visual Psychophysics* (eds. Alpern, M. et al.) 455–469 (Springer, Berlin, Heidelberg, 1972).

[27] Berry, M. H. et al. Restoration of high-sensitivity and adapting vision with a cone opsin. *Nat. Commun.* **10**, 1221 (2019). 30874546 10.1038/s41467-019-09124-x PMC6420663

[28] Berry, M. H. et al. Restoration of patterned vision with an engineered photoactivatable G protein-coupled receptor. *Nat. Commun.* **8**, 1862 (2017). 29192252 10.1038/s41467-017-01990-7 PMC5709376

