# Supplementary figures and images for: Optogenetic restoration of high-sensitivity vision using ChRmine- and ChroME-based channelrhodopsins
Source: Sci Rep. 2025 Jul 1;15:21204. doi: 10.1038/s41598-025-04286-9 (PMC12217448; doi:10.1038/s41598-025-04286-9)

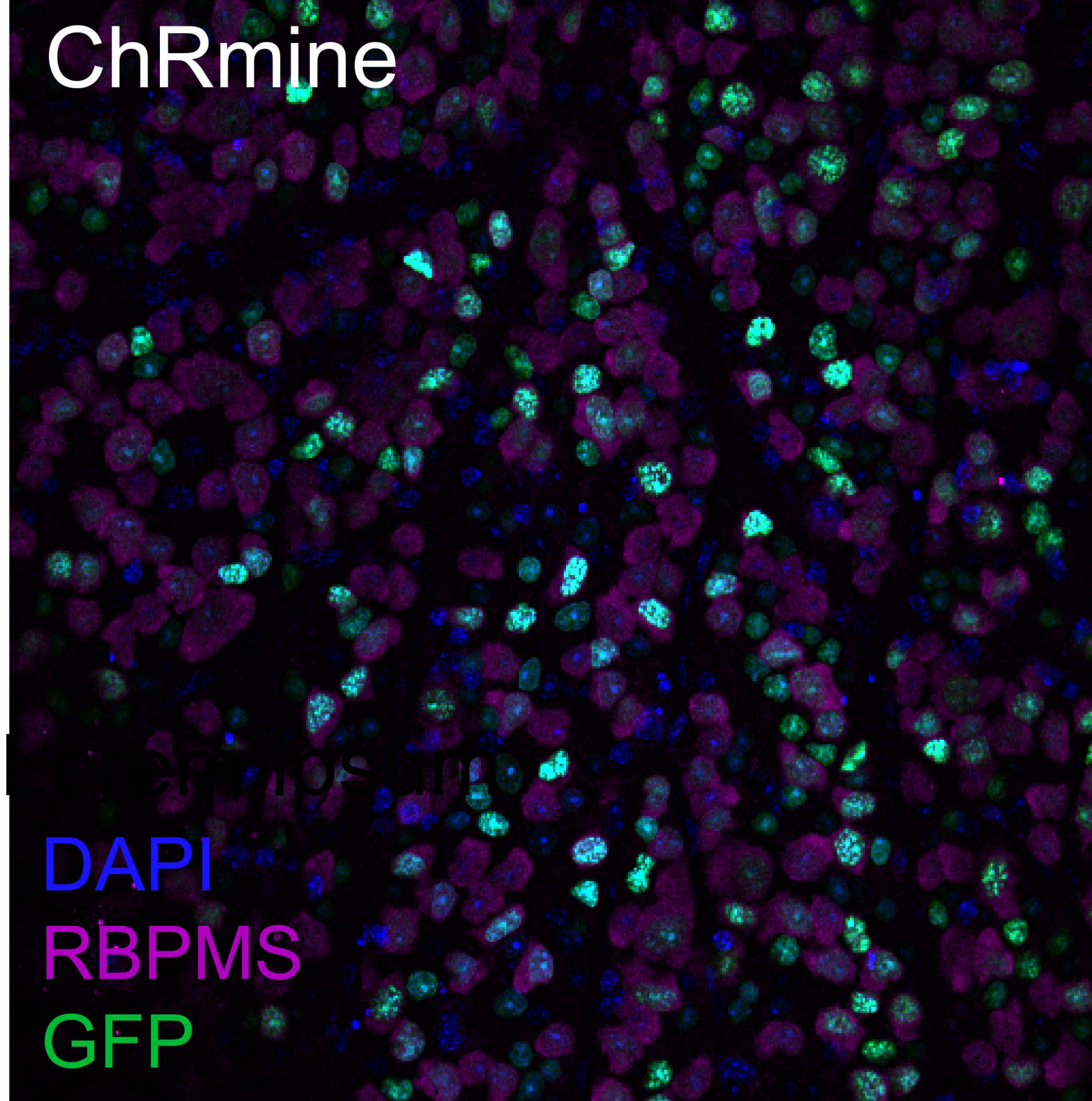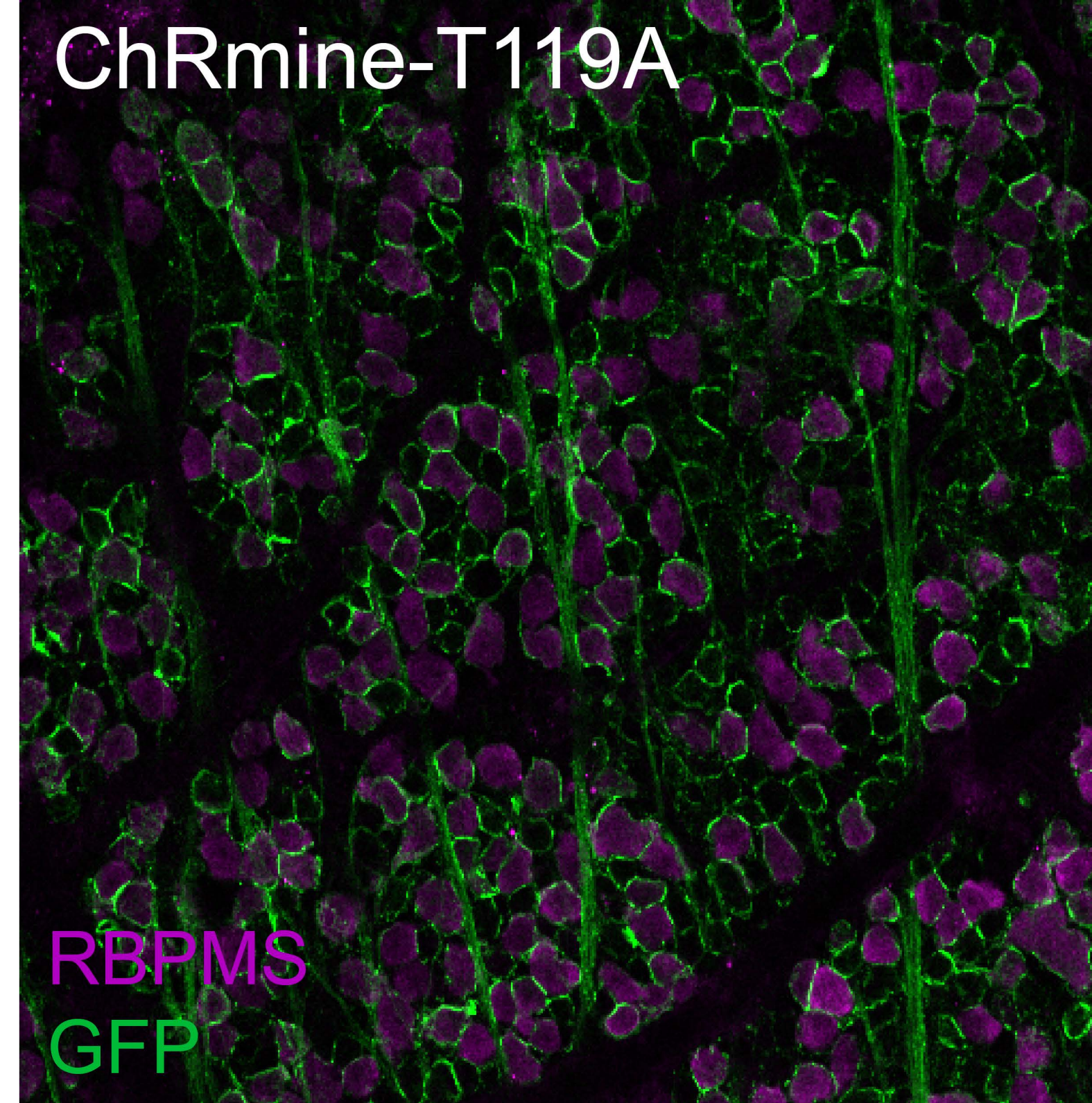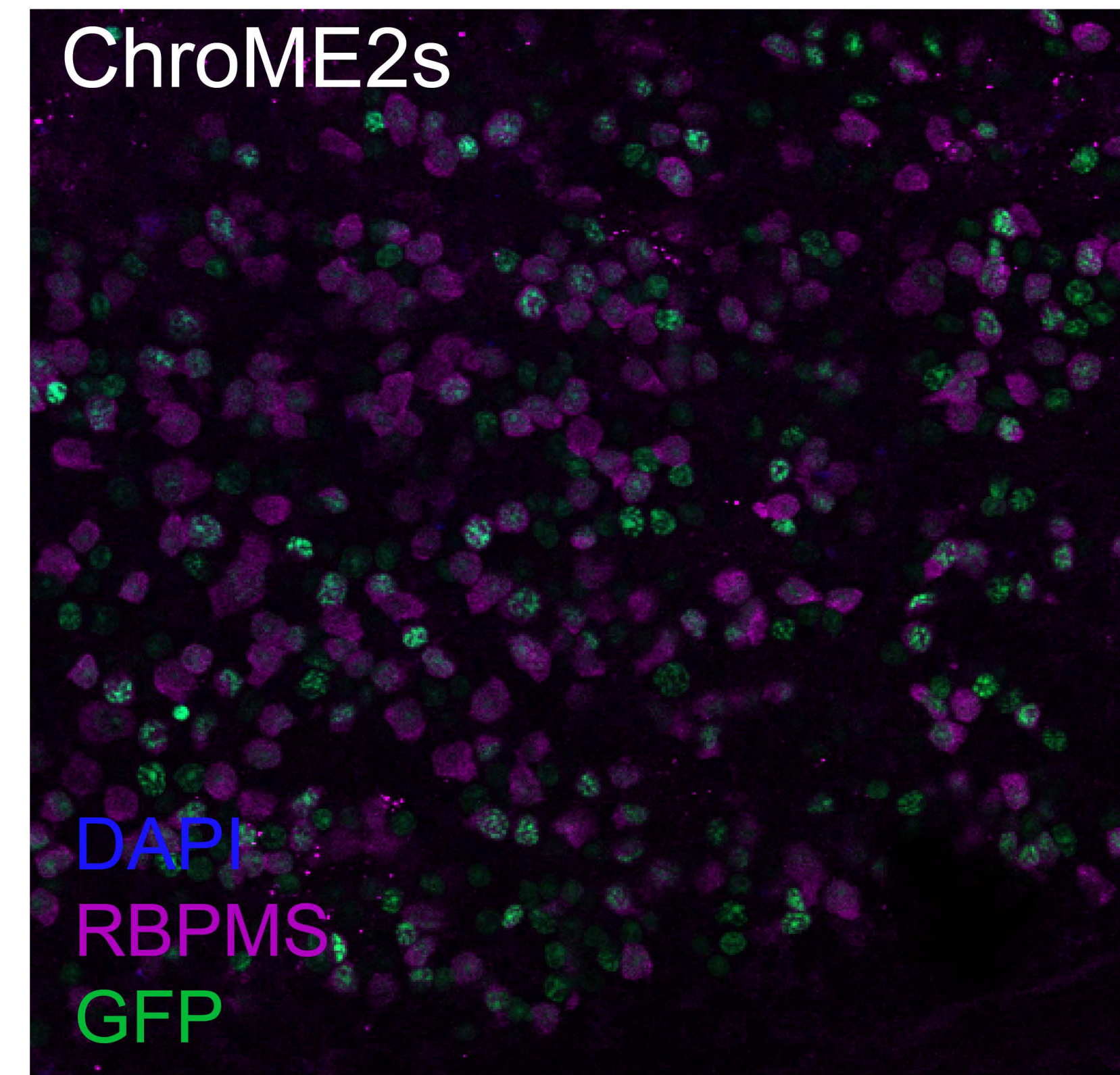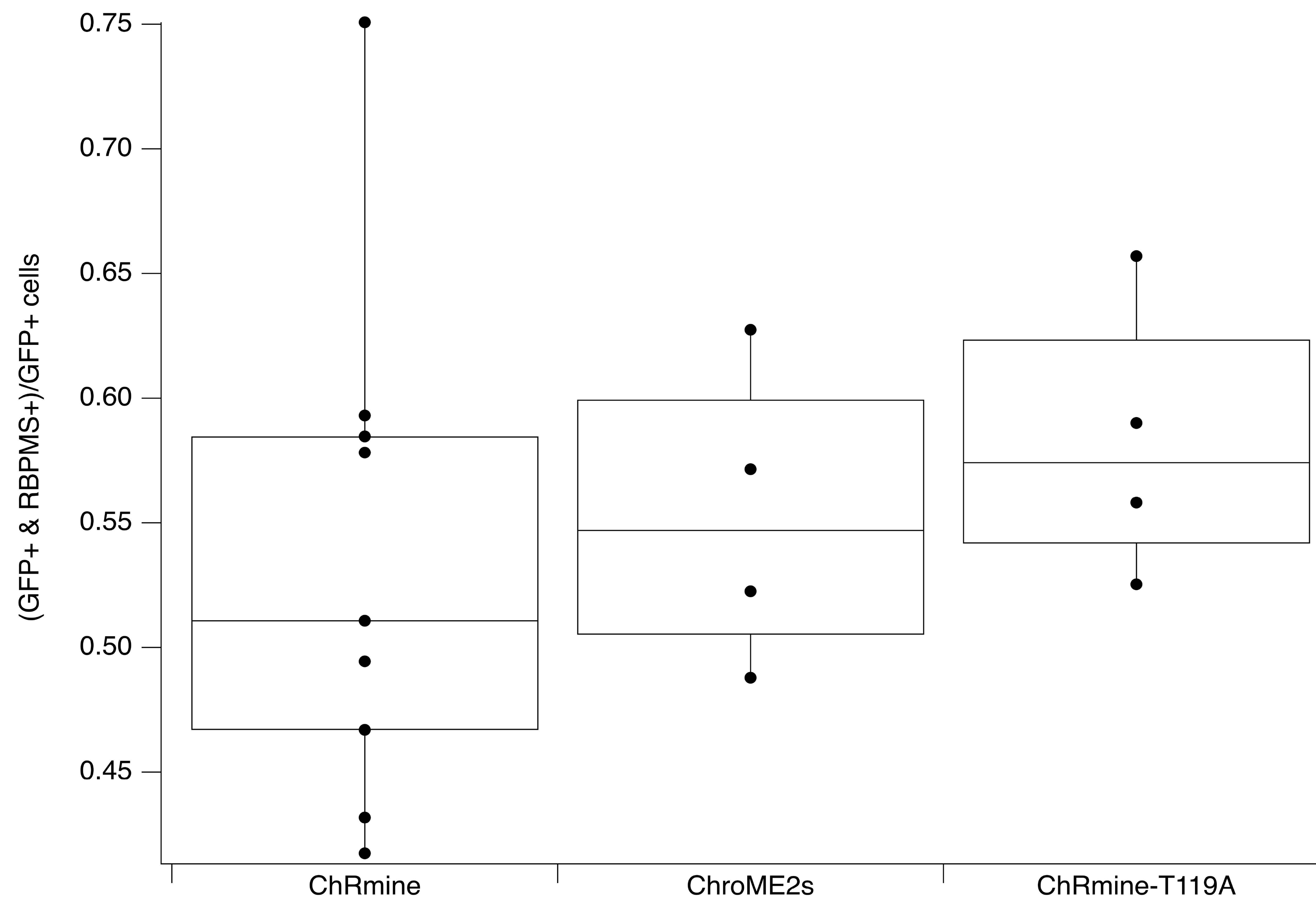

Supplement: Supplementary file 1 — Supplementary Information 1. [file 41598_2025_4286_MOESM1_ESM.pdf]

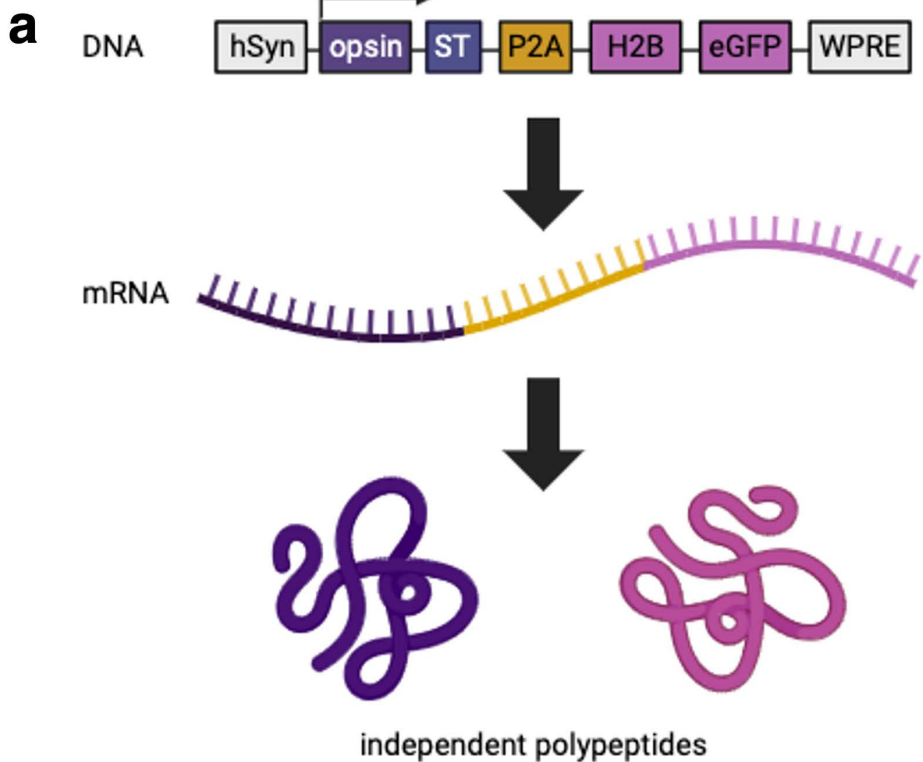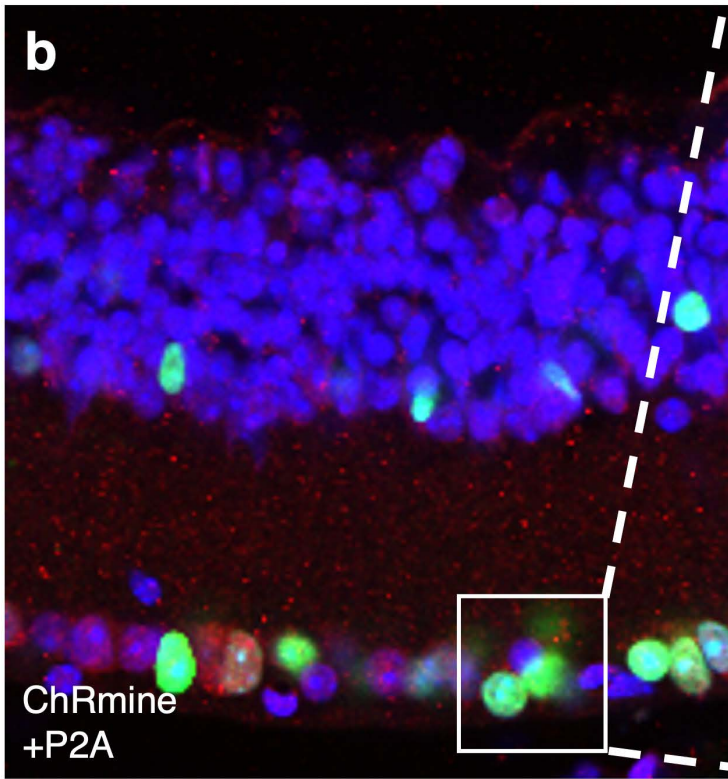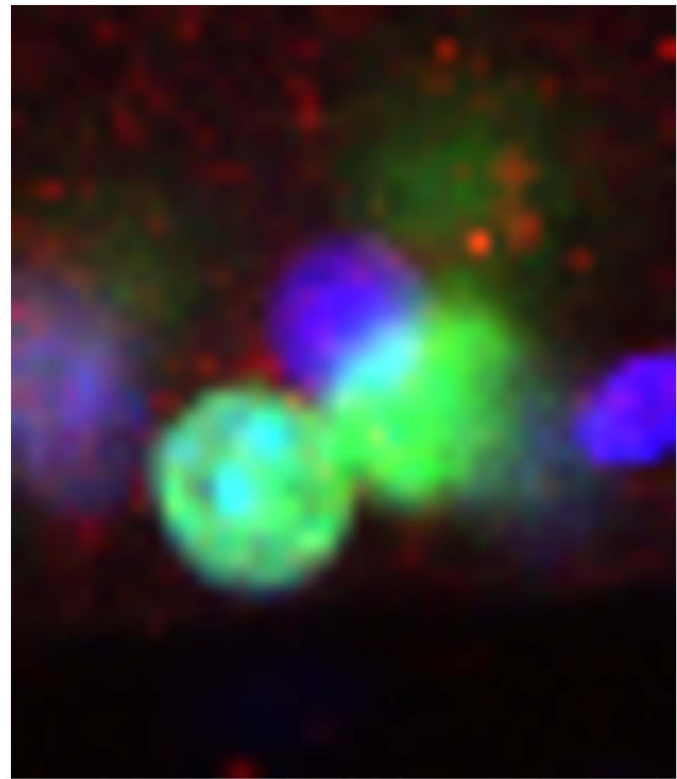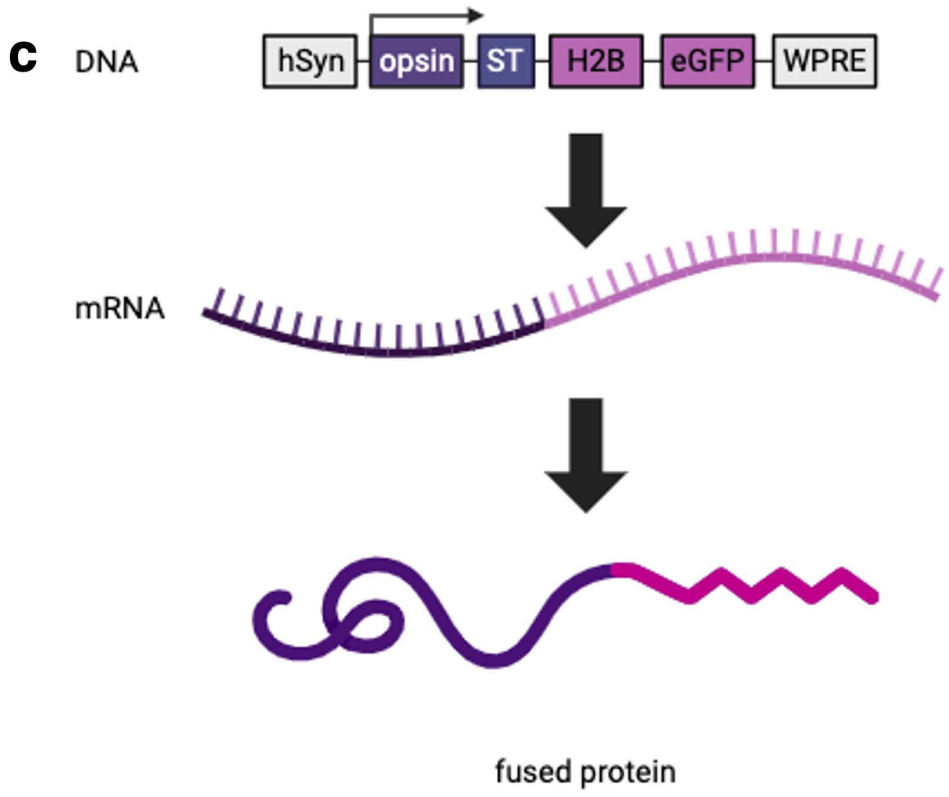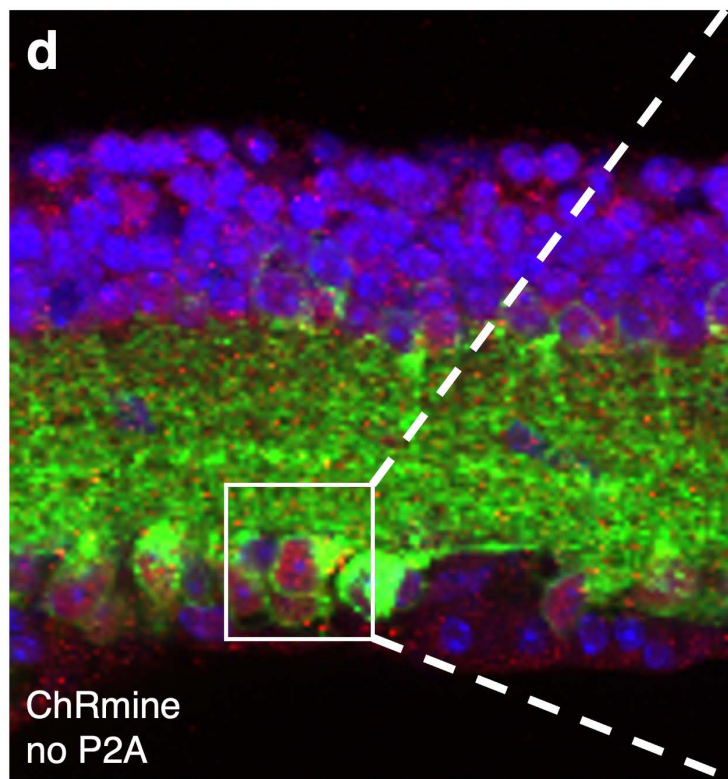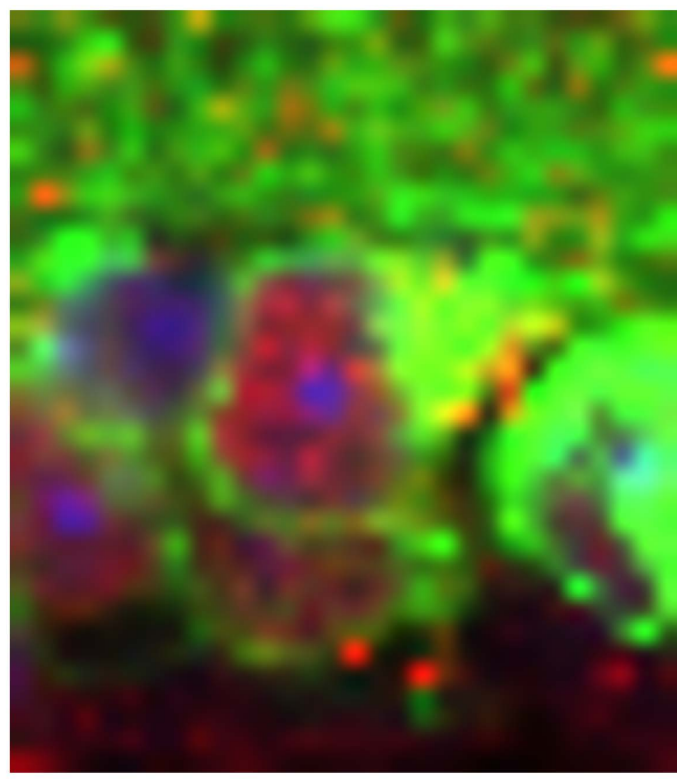

Supplement: Supplementary file 2 — Supplementary Information 2. [file 41598_2025_4286_MOESM2_ESM.pdf]

ChRmine

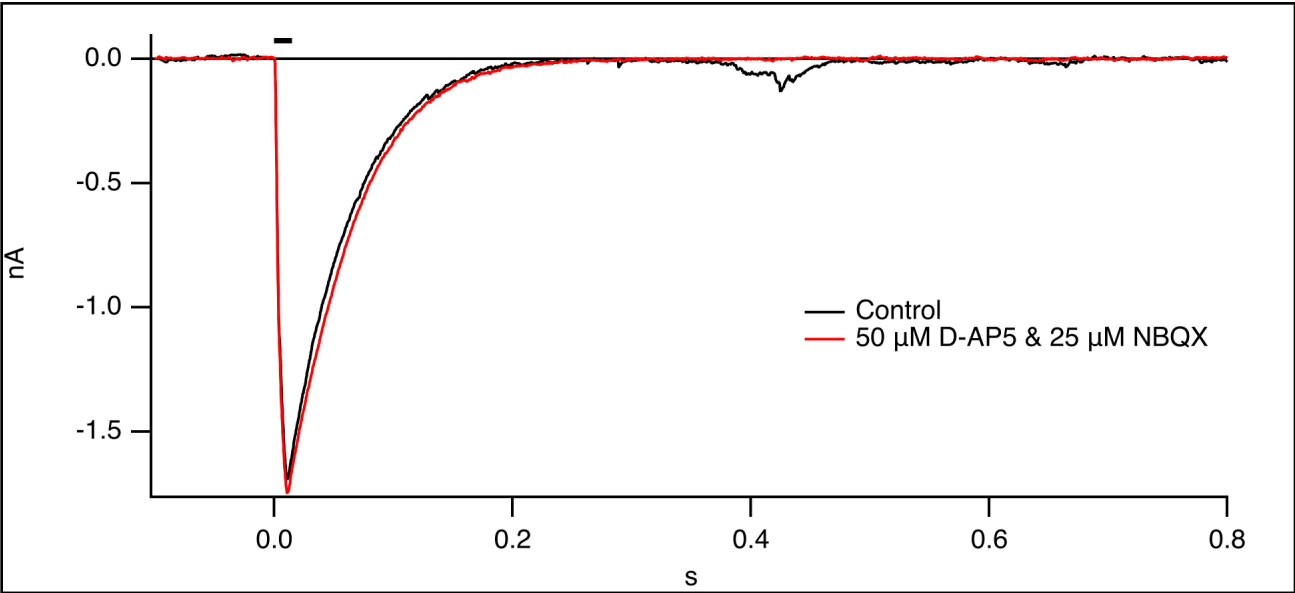

ChRmine-T119A

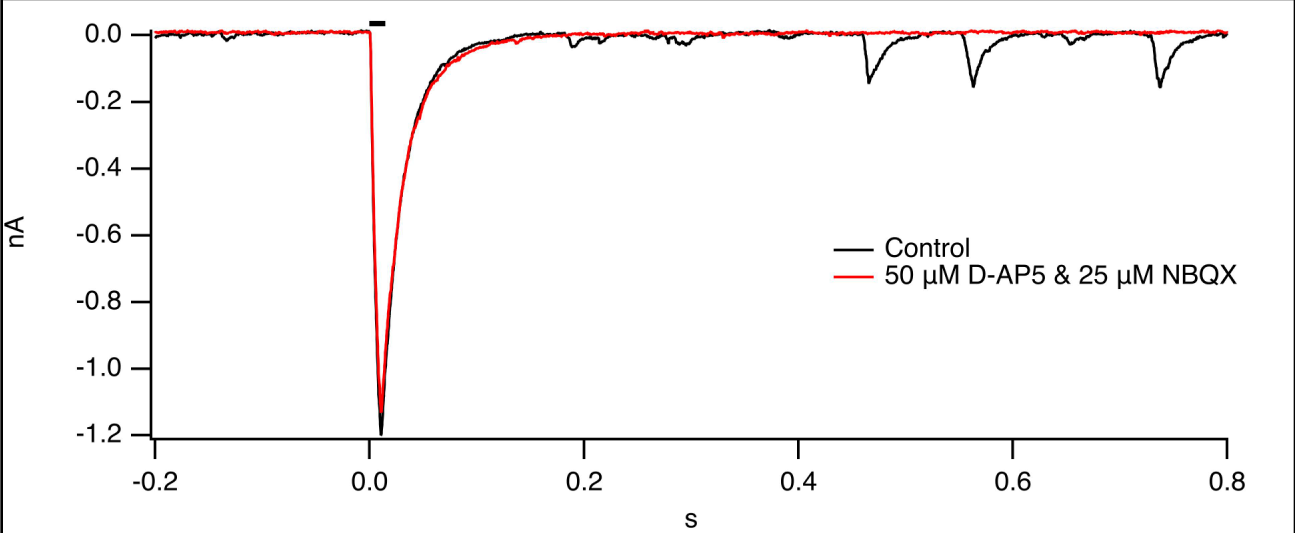

ChroME2s

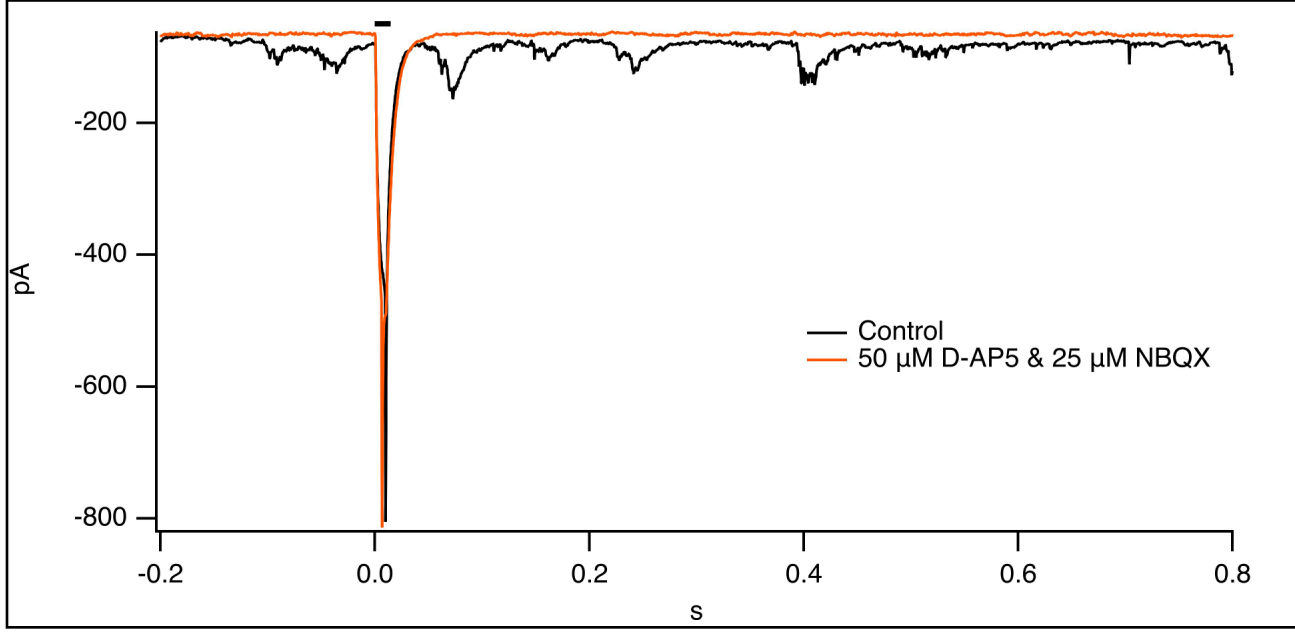

Supplement: Supplementary file 4 — Supplementary Information 4. [file 41598_2025_4286_MOESM4_ESM.pdf]

ChRmine

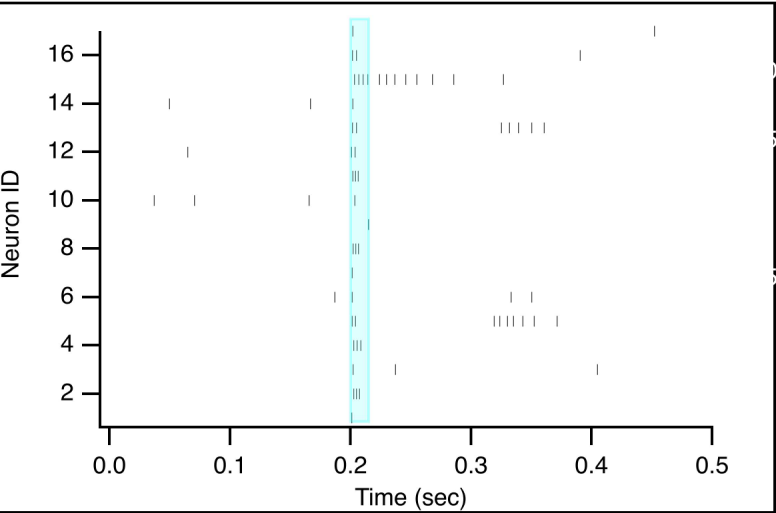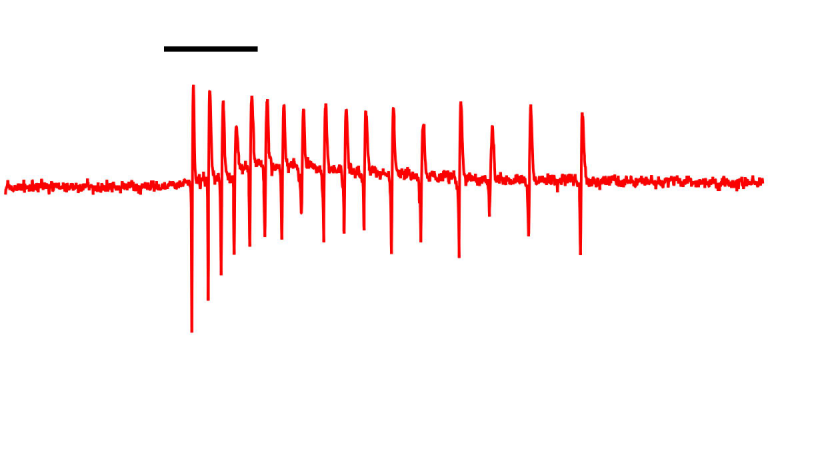

ChRmine-T119A

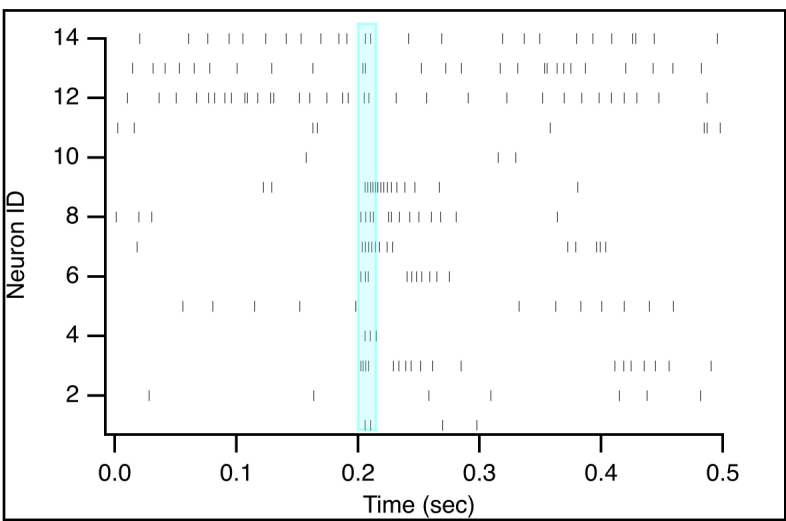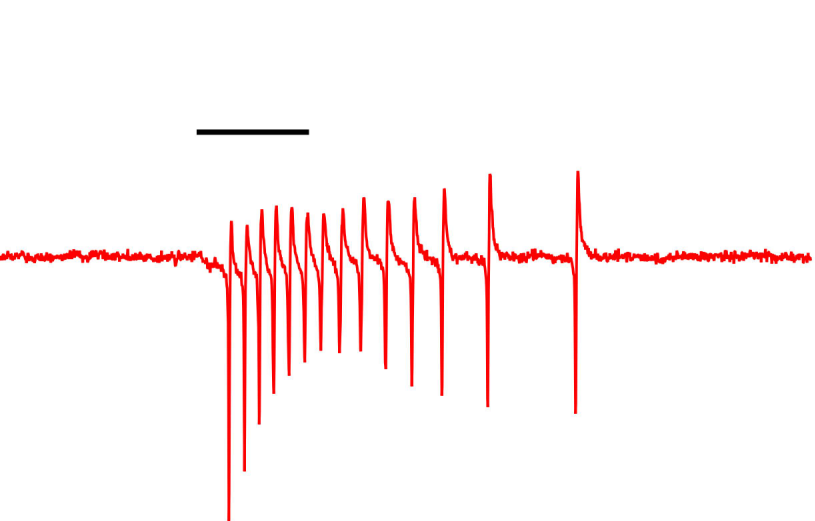

ChroME2s

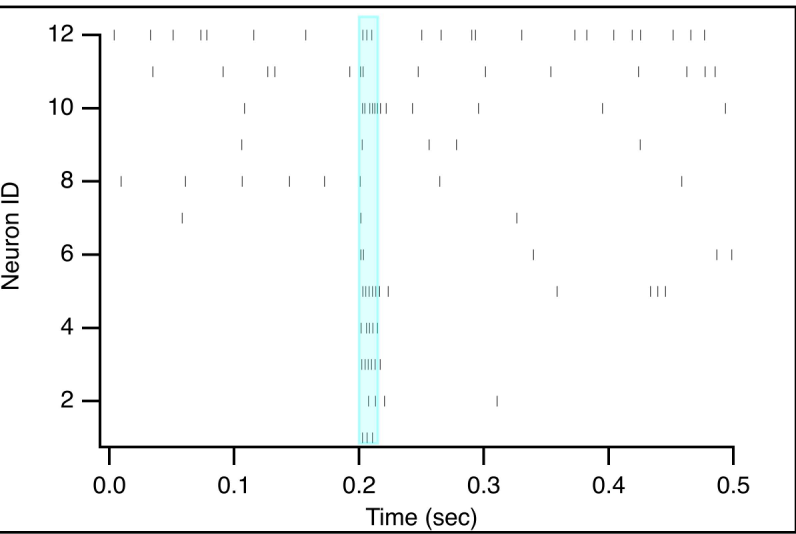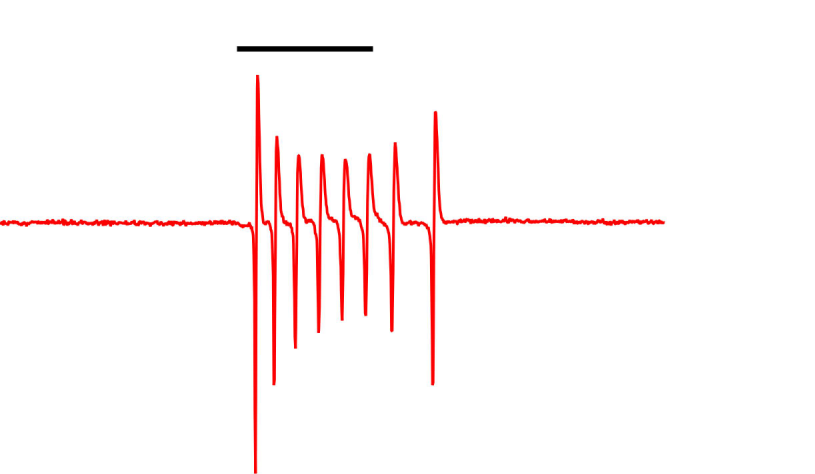

Supplement: Supplementary file 5 — Supplementary Information 5. [file 41598_2025_4286_MOESM5_ESM.pdf]

ChroME2s

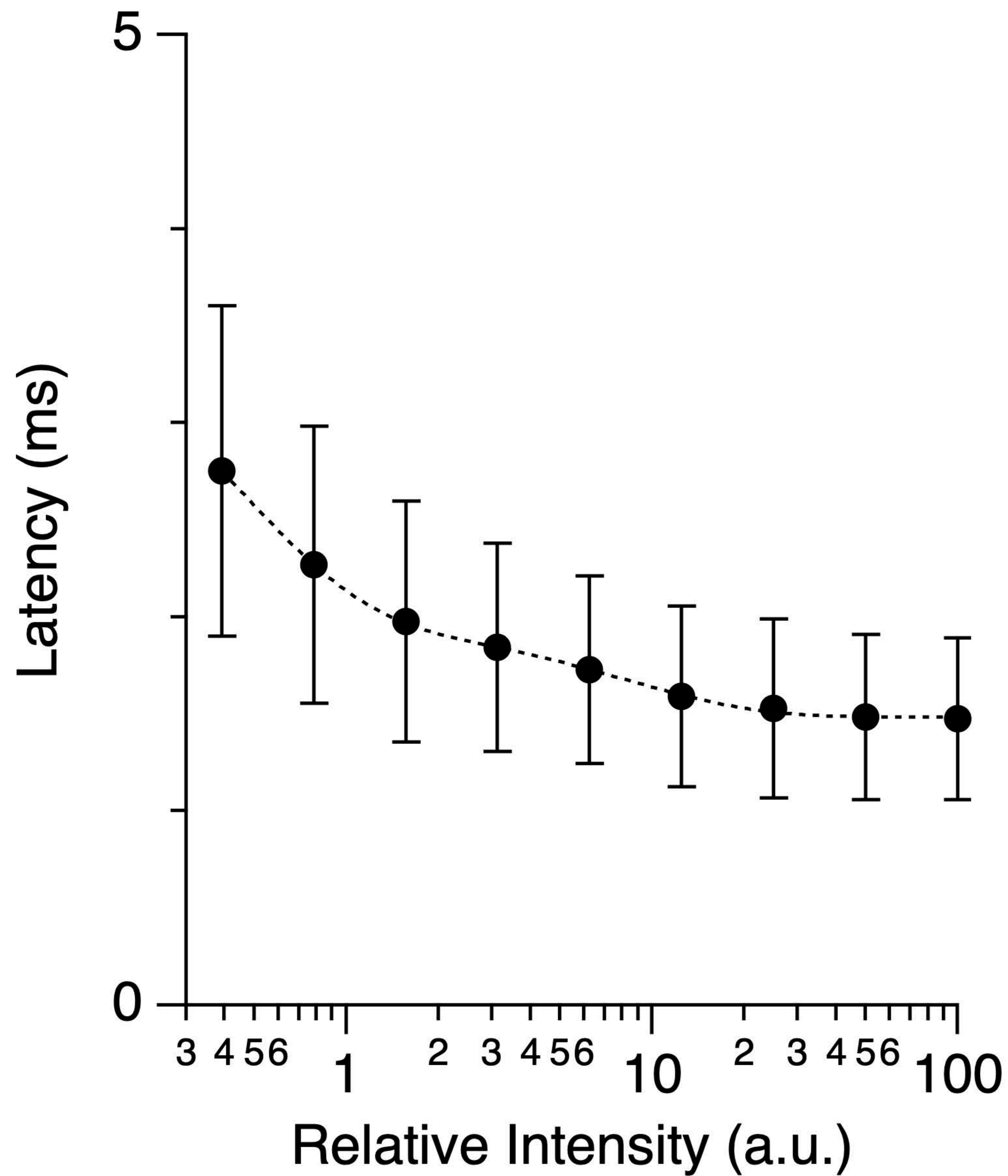

ChRmine

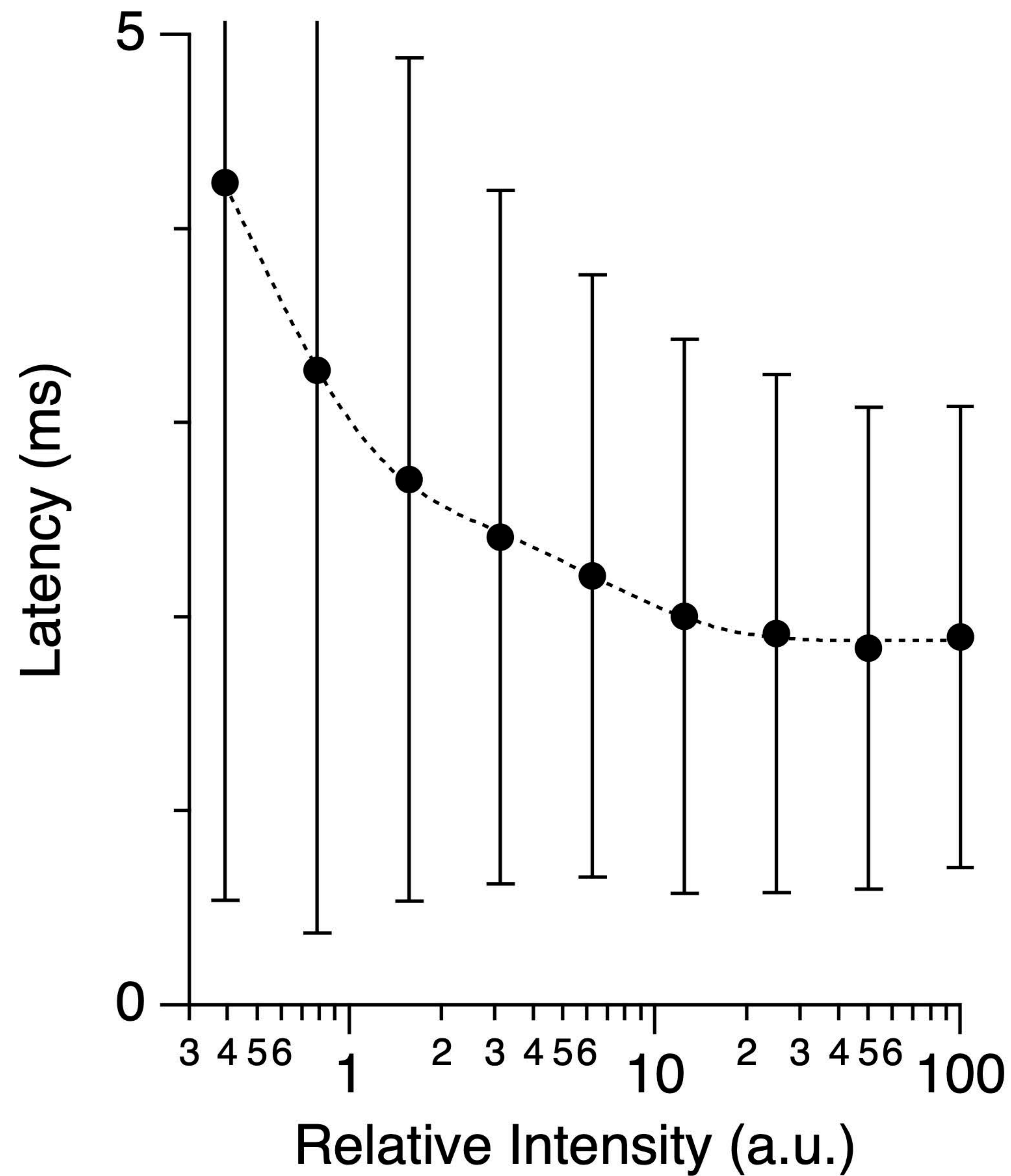

Supplement: Supplementary file 6 — Supplementary Information 6. [file 41598_2025_4286_MOESM6_ESM.pdf]

**a**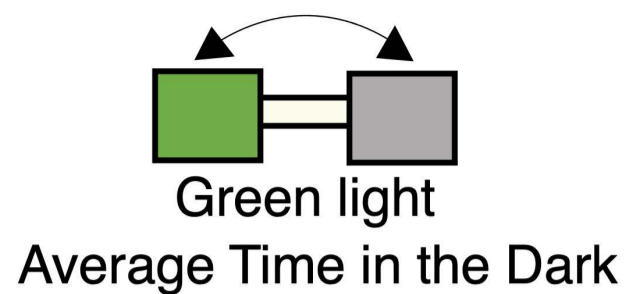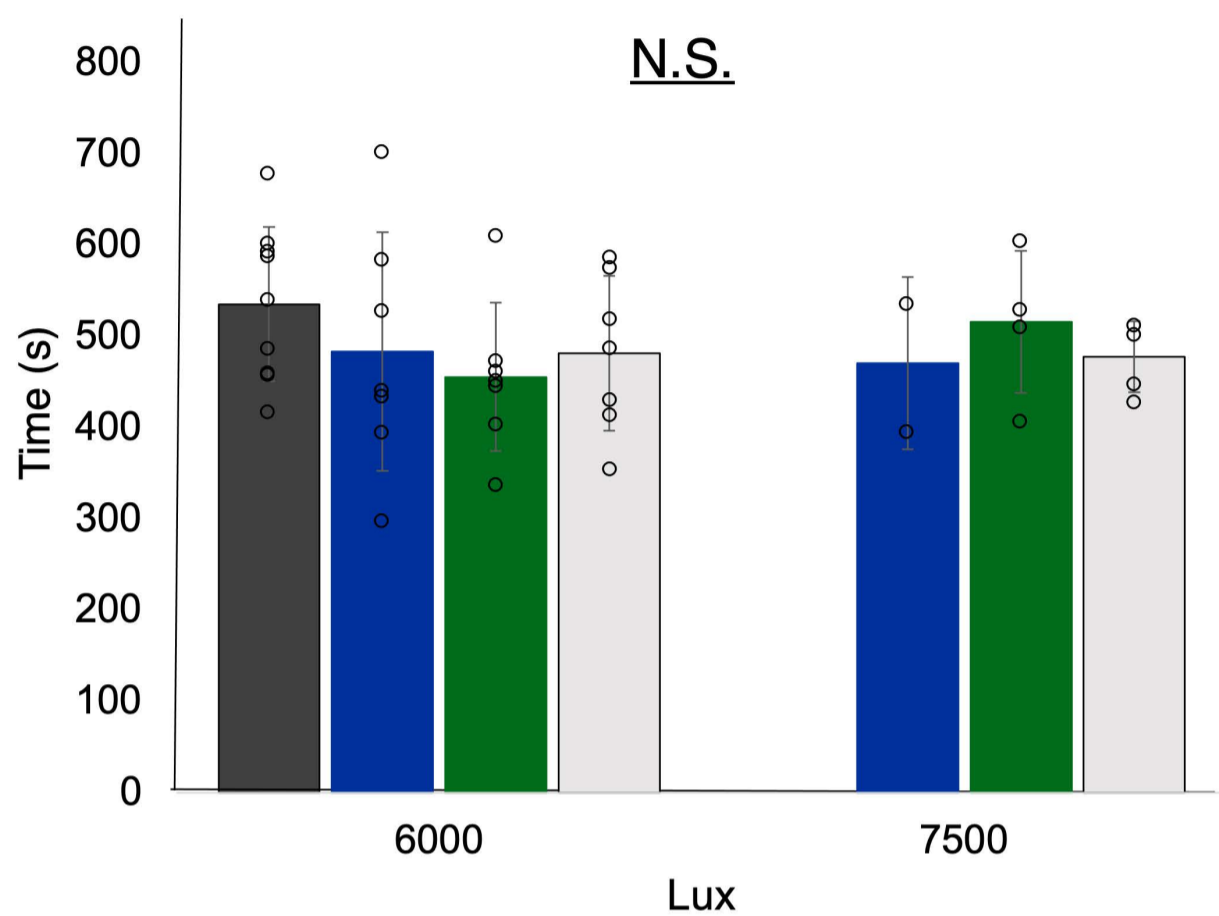**b**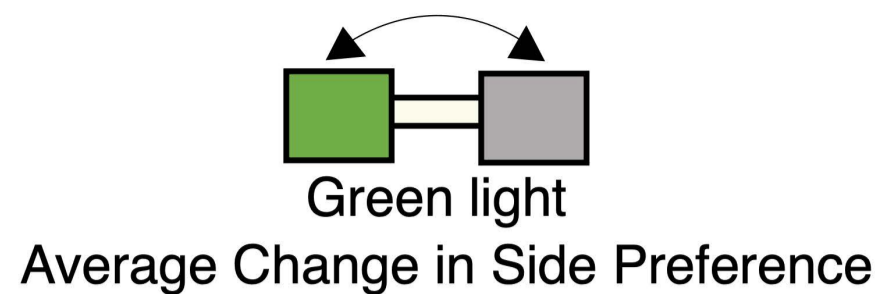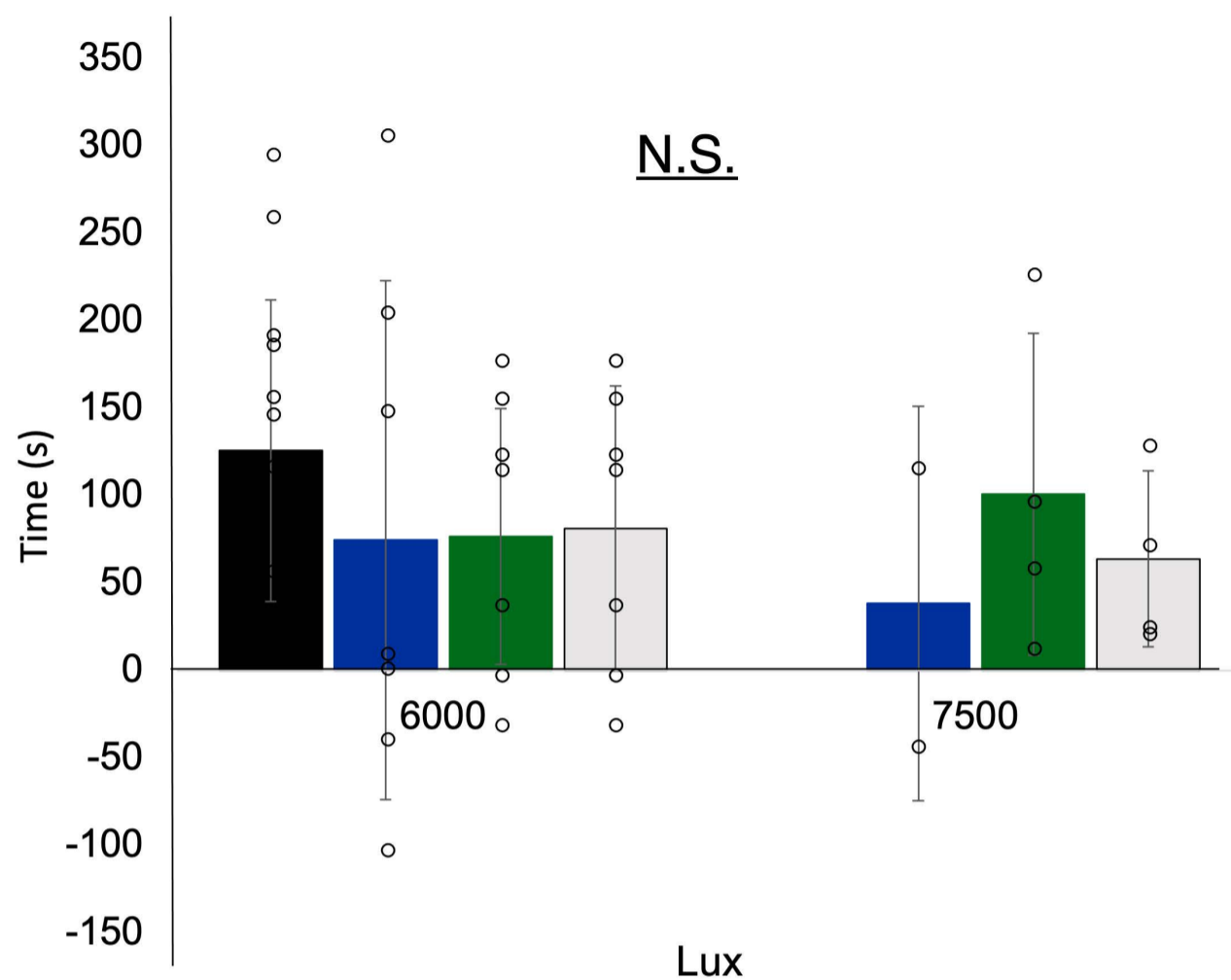**c**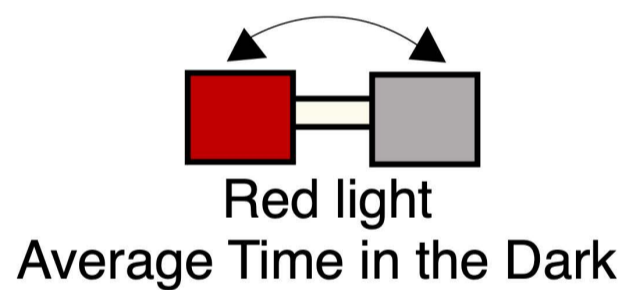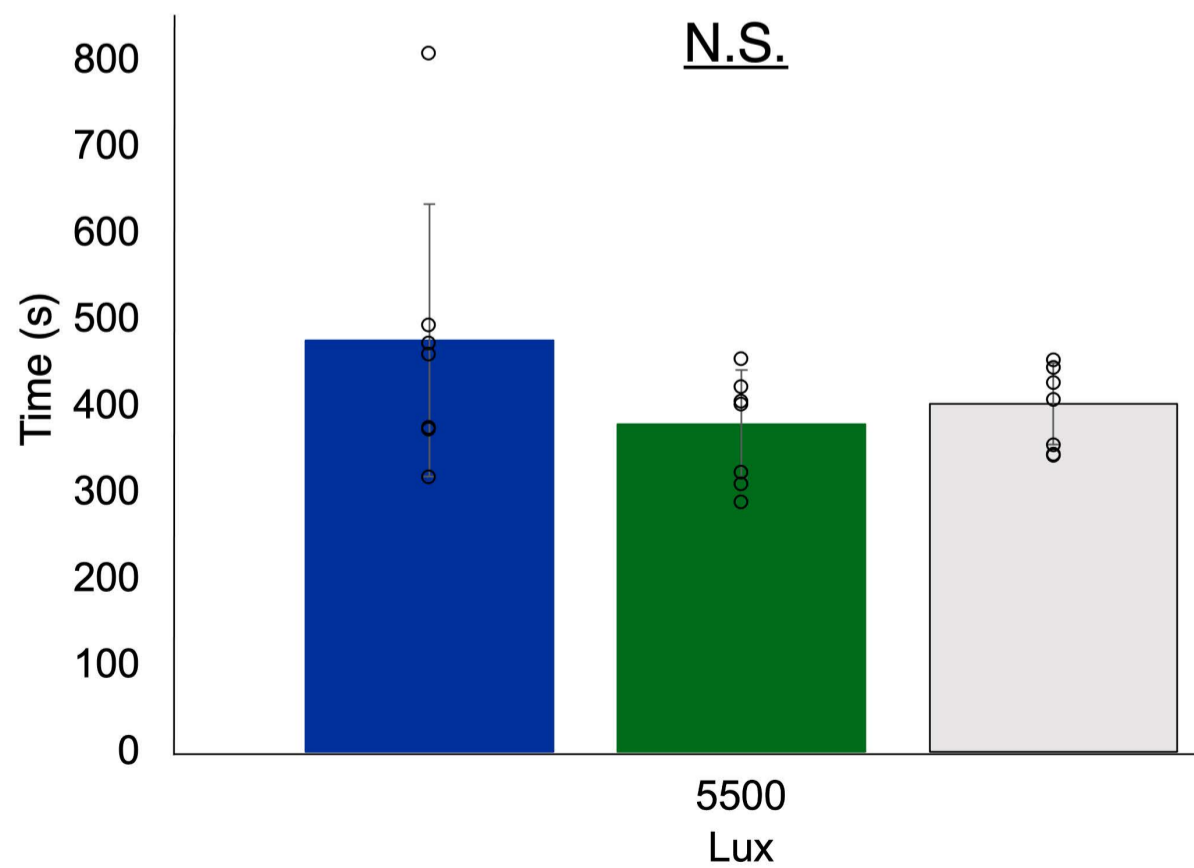**d**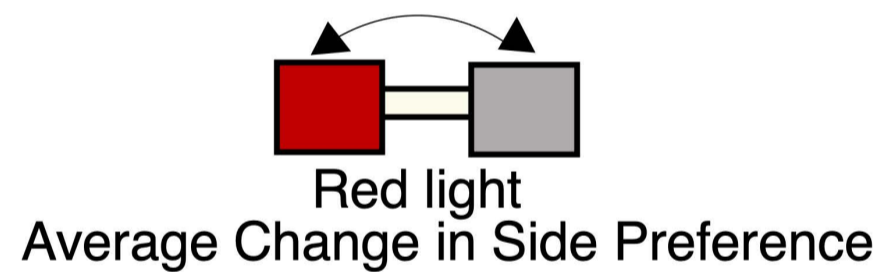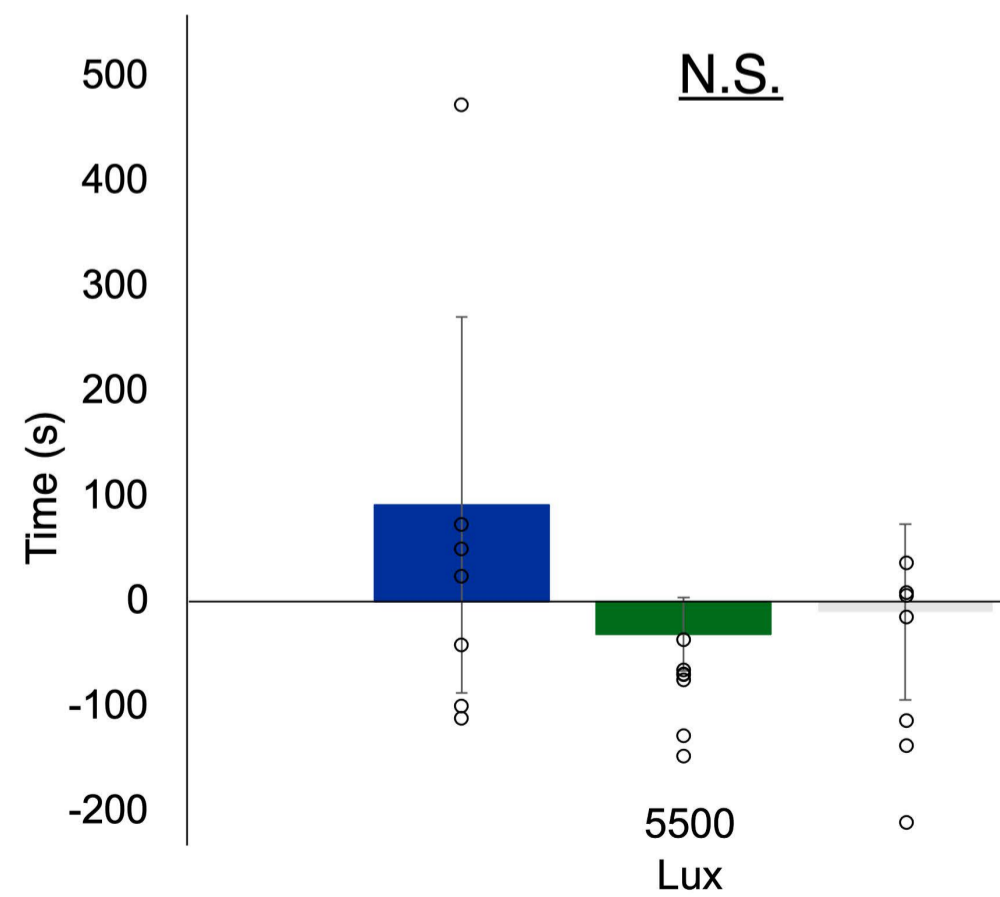

■ C57BL6/J

■ ChRmine-ST  
8.53E+14vg/ml■ ChroME2S-ST  
1.515E+15vg/ml

■ PBS-sham

Supplement: Supplementary file 7 — Supplementary Information 7. [file 41598_2025_4286_MOESM7_ESM.pdf]

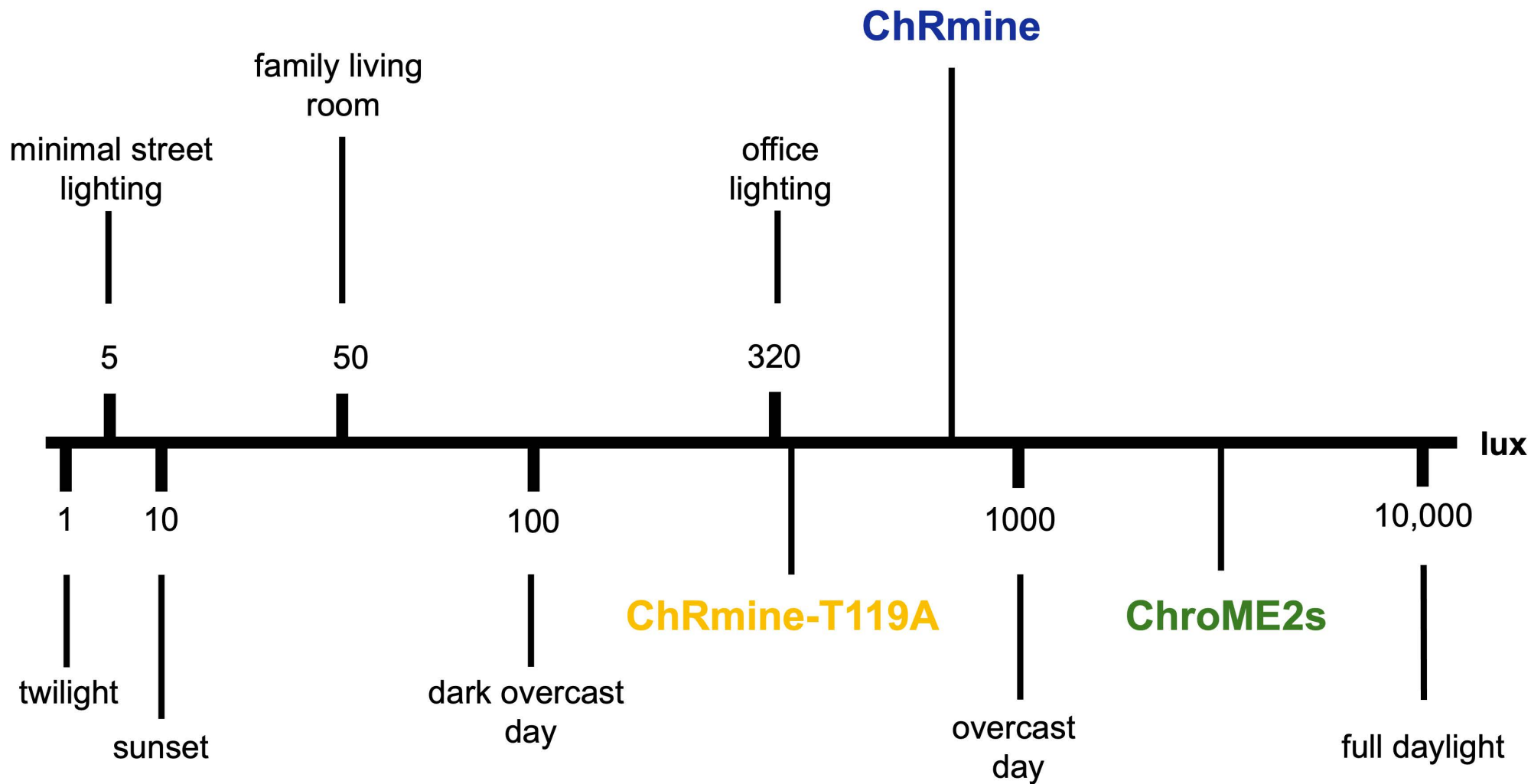

Supplement: Supplementary file 8 — Supplementary Information 8. [file 41598_2025_4286_MOESM8_ESM.pdf]
