# Supplementary material for: Optogenetic restoration of high-sensitivity vision using ChRmine- and ChroME-based channelrhodopsins
Source: Sci Rep. 2025 Jul 1;15:21204. doi: 10.1038/s41598-025-04286-9 (PMC12217448; doi:10.1038/s41598-025-04286-9)
Supplement: Supplementary file 3 — Supplementary Information 3. [file 41598_2025_4286_MOESM3_ESM.pdf]

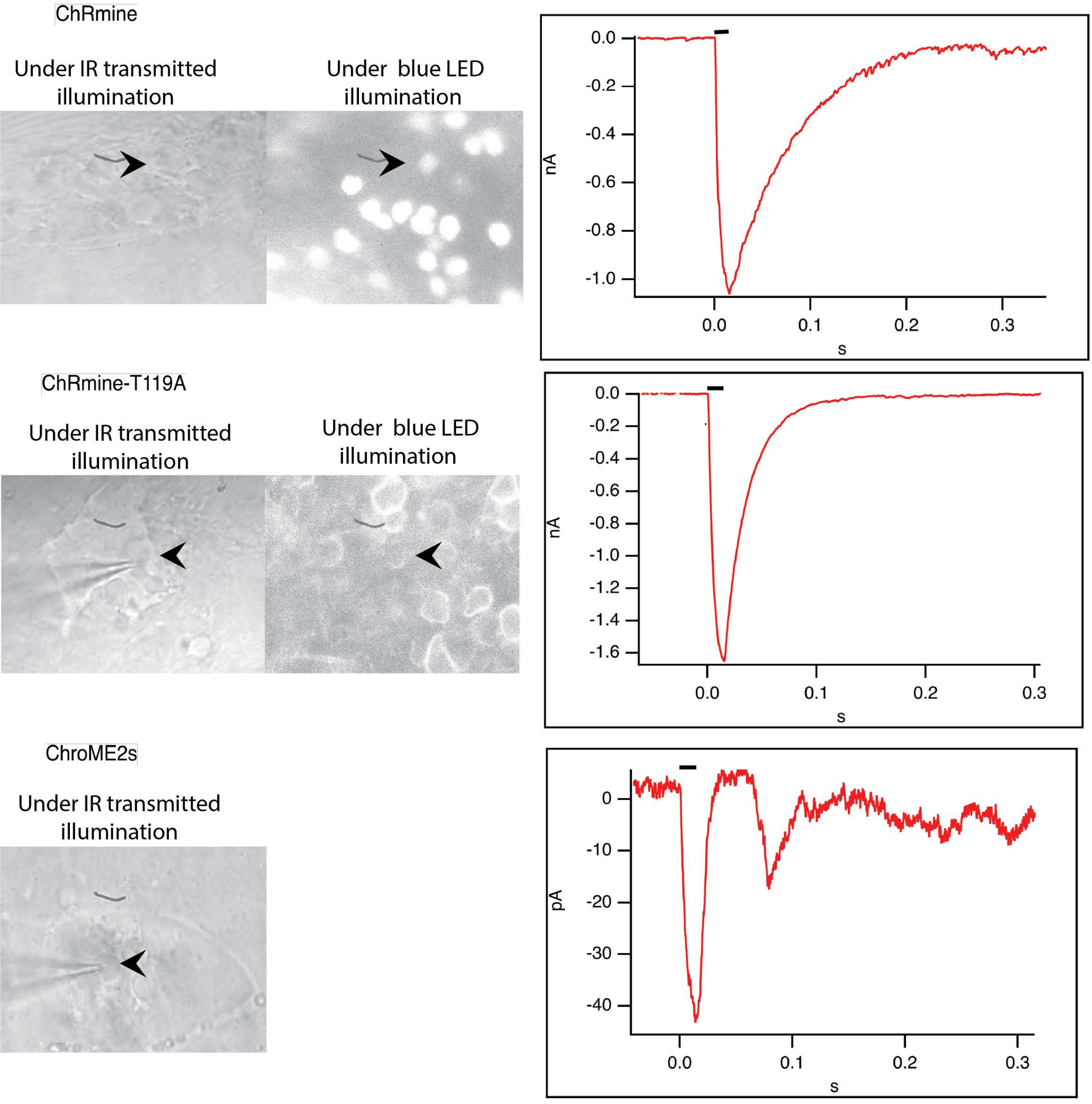
ChRmine
Under IR transmitted illumination
Under blue LED illumination
nA
s
0.0
-0.2
-0.4
-0.6
-0.8
-1.0
0.0
0.1
0.2
0.3
ChRmine-T119A
Under IR transmitted illumination
Under blue LED illumination
nA
s
0.0
-0.2
-0.4
-0.6
-0.8
-1.0
-1.2
-1.4
-1.6
0.0
0.1
0.2
0.3
ChroME2s
Under IR transmitted illumination
pA
s
0
-10
-20
-30
-40
0.0
0.1
0.2
0.3
